# Characterization of an archaeal virus-host system reveals massive genomic rearrangements in a laboratory strain

**DOI:** 10.3389/fmicb.2023.1274068

**Published:** 2023-09-18

**Authors:** Coraline Mercier, Daniela Thies, Ling Zhong, Mark J. Raftery, Susanne Erdmann

**Affiliations:** ^1^Max Planck Institute for Marine Microbiology, Archaeal Virology, Bremen, Germany; ^2^Bioanalytical Mass Spectrometry Facility, The University of New South Wales, Sydney, NSW, Australia; ^3^School of Biotechnology and Biomolecular Sciences, The University of New South Wales, Sydney, NSW, Australia

**Keywords:** *Halorubrum lacusprofundi*, secondary chromosome, haloarchaea, archaeal virus, virus defense, genome plasticity

## Abstract

Halophilic archaea (haloarchaea) are known to exhibit multiple chromosomes, with one main chromosome and one or several smaller secondary chromosomes or megaplasmids. *Halorubrum lacusprofundi*, a model organism for studying cold adaptation, exhibits one secondary chromosome and one megaplasmid that include a large arsenal of virus defense mechanisms. We isolated a virus (Halorubrum tailed virus DL1, HRTV-DL1) infecting *Hrr. lacusprofundi*, and present an in-depth characterization of the virus and its interactions with *Hrr. lacusprofundi*. While studying virus-host interactions between *Hrr. lacusprofundi* and HRTV-DL1, we uncover that the strain in use (ACAM34_UNSW) lost the entire megaplasmid and about 38% of the secondary chromosome. The loss included the majority of virus defense mechanisms, making the strain sensitive to HRTV-DL1 infection, while the type strain (ACAM34_DSMZ) appears to prevent virus replication. Comparing infection of the type strain ACAM34_DSMZ with infection of the laboratory derived strain ACAM34_UNSW allowed us to identify host responses to virus infection that were only activated in ACAM34_UNSW upon the loss of virus defense mechanisms. We identify one of two S-layer proteins as primary receptor for HRTV-DL1 and conclude that the presence of two different S-layer proteins in one strain provides a strong advantage in the arms race with viruses. Additionally, we identify archaeal homologs to eukaryotic proteins potentially being involved in the defense against virus infection.

## Introduction

Secondary chromosomes and megaplasmids are additional chromosomes that coexist alongside the primary circular chromosome, and are found in bacteria and archaea (Harrison et al., 2010; Hall et al., 2022). Amongst archaea, halophilc archaea belonging to the Euryarchaeota (haloarchaea) are particularly rich in additional chromosomes (DasSarma et al., 2009). While the exact number of additional chromosomes can vary between different species, it is not uncommon for haloarchaea to possess two or more of these extra genetic elements (DasSarma et al., 2009; Wang et al., 2015). Secondary chromosomes, are distinguished from megaplasmid by carrying essential housekeeping genes (Hall et al., 2022). While some of the additional chromosomes in haloarchaea strongly interact with the main chromosomes and exhibit some essential genes (Harrison et al., 2010; Wang et al., 2015), others have been lost and shown to be non-essential (Hawkins et al., 2013). *Hrr. lacusprofundi* exhibits three replicons, the main chromosome CHR1, a secondary chromosome CHR2 and the megaplasmid pHLAC01. A metagenomics study revealed that the main chromosome of *Hrr. lacusprofundi* is highly conserved between strains, but the secondary chromosomes and megaplasmids are very diverse and responsible for the genetic diversity of the species (Tschitschko et al., 2018).

Haloarchaea are infected by diverse viruses including head-tailed viruses, pleomorphic viruses, spherical viruses, and spindle shaped viruses (Luk et al., 2014). A number of haloarchaeal viruses exhibit lysogenic and chronic life cycles, however, the majority of isolated viruses exhibit lytic life cycles, reflecting a methodical bias caused by using plaque assays for isolation, selecting mostly for lytic viruses (Alarcón-Schumacher et al., 2022). A recent study described a number of new haloarchaeal tailed viruses and proposed a new classification of archaeal tailed viruses (arTV) (Liu Y. et al., 2021). The majority of haloarchaeal tailed viruses, exhibiting major capsid proteins (MCPs) with the HK97 fold, belong to the “myovirus” or “siphovirus” morphotype, only one isolate exhibits “podovirus” morphology (Pietilä et al., 2013b). While all package a linear genome with size ranging from 26 kb to 143 kb, a few exhibit circular permuted ends and the majority exhibit direct terminal repeats ranging in size from 229 bp up to 739 bp (Liu Y. et al., 2021). The host range of haloarchaeal tailed viruses appears to be very variable, with some exhibiting a very broad host range and some being very host specific (Atanasova et al., 2012). While it is still unclear how arTVs exit their host cells, the S-layer has recently been identified as possible receptor for a haloarchaeal tailed virus (Schwarzer et al., 2023).

Even though arTV appear morphological similar to head-tailed bacteriophages, they encounter cells that are dramatically different, in particular with respect to the cell surface, and replication, transcription and translation mechanisms. While the differences between bacterial and archaeal head-tailed viruses is clearly reflected by genomic differences, the effect of these differences on virus-host interactions remains severely understudied. To date only very few arTV infecting haloarchaea have been characterized in more depth (Luk et al., 2014; Liu et al., 2021).

Archaea use primarily the CRISPR-Cas (Clustered Regularly Interspaced Short Palindromic Repeats and CRISPR-associated genes) system, Toxin-Antitoxin (TA) systems, and Restriction Modification (RM) systems, additional to changes of cell surface proteins, to defend against viruses (Koonin et al., 2017). The majority of haloarchaea exhibit CRISPR-Cas systems, RM systems and TA systems, and CRISPR systems have been experimentally shown to be active against extrachromosomal elements in haloarchaea (Li et al., 2014; Maier et al., 2019). Argonaut proteins, known to be important for virus defense in eukaryotes (Obbard et al., 2009), have also been identified on archaeal genomes (Willkomm et al., 2018). However, while DNA-targeting activity has been shown experimentally for an archaeal Argonaut protein (Zander et al., 2017), evidence for exclusion of extrachromosomal elements by archaeal Argonauts is pending. Recent studies, using bioinformatics approaches, identified an enormous diversity of previously unrecognized bacterial virus exclusion mechanisms (Bernheim and Sorek, 2020; Millman et al., 2022), of which some were also detected in archaea. For example, CBASS (Cyclic oligonucleotide-based antiphage signaling system) systems were detected in a number of archaeal genomes and were shown to block virus propagation in bacteria by inducing cell death of the infected cell (Duncan-Lowey and Kranzusch, 2022). The BREX (Bacteriophage Exclusion) system, also discovered on archaeal genomes, was shown to block virus replication (Goldfarb et al., 2015). While a Dnd defense system has recently been experimentally investigated in archaea (Xiong et al., 2019), the majority of the newly discovered defense systems that are not as widespread amongst archaea as CRISPR-Cas, TA or RM systems, remain uncharacterised.

Here we present an in-depth characterization of the interactions of an archaeal tailed virus and its host, including the characterization of the virus life cycle, determination of the virus host range, analysis of the host transcriptional response to virus infection and the characterization of virus escape mutants. We uncover that the host strain in use lost an entire megaplasmid and a part of its secondary chromosome while being grown under laboratory conditions. This loss included the majority of virus defense systems, including a CRISPR-Cas system, TA and RM systems and a BREX system, which allows us to discover new putative virus defense mechanisms and the host receptor for virus attachment.

## Results and discussion

### Isolation of a lytic virus that infects *Halorubrum lacusprofundi* ACAM34 (DSM 5036)

**Figure 1:**
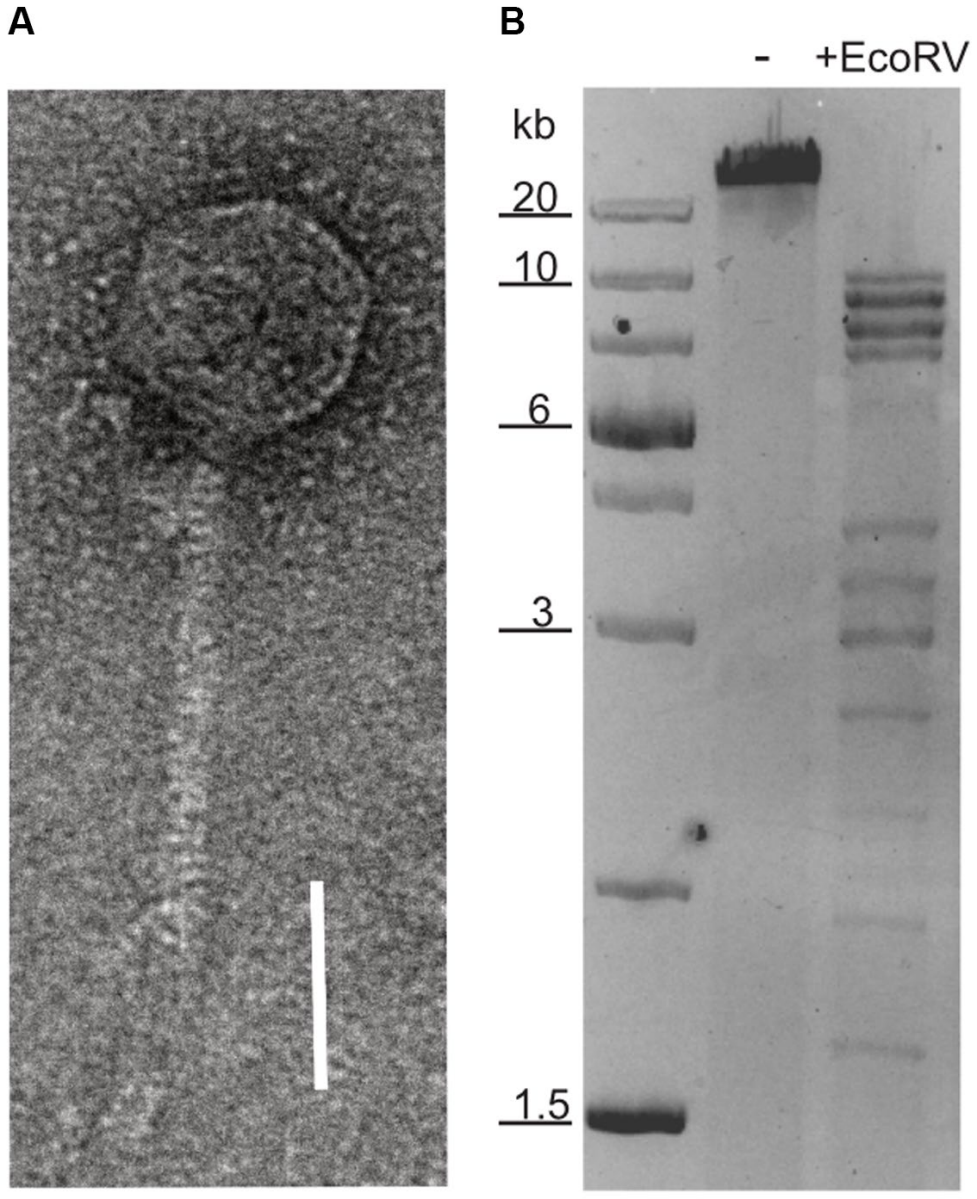
Nucleic acid content and particle of HRTV-DL. **(A)** Electron micrograph of HRTV-DL particle. Sample was negatively stained with 2% uranyl acetate. Size bar: 50 nm. **(B)** Total DNA of HRTV-DL undigested (-) and digested with EcoRV (+EcoRV). MW size marker is shown to the left of the gel (GeneRuler 1 kb Plus DNA Ladder, Thermo Fisher Scientific). DNA was separated on 1% agarose gels and stained with SYBR™ Safe DNA stain. Size bar: 50 nm. Original images have been modified by cropping to improve visual presentation.

A virus with head-tailed morphology (Figure 1A) was obtained from the culture supernatant of a *Halorubrum lacusprofundi* strain, that was isolated from a sample taken in 2014 from Deep Lake, Antarctica (Gibson, 1999). Analytic restriction digest of DNA isolated from the viral lysate revealed a dsDNA genome of approximately 60 kb (Figure 1B). The lysate produced plaques on *Hrr. lacusprofundi* strain ACAM34 (Franzmann et al., 1988) that was isolated from a Deep Lake sample in 1988, while it remains elusive when the sample was actually obtained from Deep Lake. Lysis was also observed in liquid cultures of this strain. The viral lysate was formerly describe as DLHTHV (Tschitschko et al., 2018), however, we rename it hereby as HRTV-DL (Halorubrum Tailed Virus-Deep Lake), according to the nomenclature that was previously used for archaeal tailed viruses (arTV) (Liu Y. et al., 2021). HRTV-DL is the first virus infecting *Hrr. lacusprofundi* that has been isolated from its original environment, Deep Lake. *Hrr. lacusprofundi* ACAM34 was used to purify the virus through several rounds of plaque assays. When analyzing genomic DNA of virus particles propagated from single plaques, we observed a remarkable high genomic variability (Supplementary Figure S1) and a reduction of the genome size. Such genomic variation, upon isolation, has previously been observed for *Halobacterium salinarium* virus Phi H (Schnabel et al., 1982) and suggests the presence of several variants in the initial HRTV-DL preparation. After two rounds of plaque purification, we chose one variant (P2V1, Supplementary Figure S1C), HRTV-DL1, for further characterization. Virus particles analyzed by electron microscopy exhibit a head-tailed morphology, with a head diameter of about 50 nm and a non-contractile tail of approximately 80 nm-100 nm in length (Figure 1B and Supplementary Figure S2).

### Genomic features of HRTV-DL1 and proteins associated with the virus particles

DNA isolated directly from HTRV-DL1 particles appeared sensitive to Exonuclease treatment, indicating a linear genome (Supplementary Figure S3). However, the majority of viral DNA isolated out of infected cells was shown to be insensitive to Exonuclease. Therefore, we suggest that the genome is replicated as a circular genome within the host and packaged as linear genome into virus particles. When comparing the EcoRV digested genome of the intracellular virus after Exonuclease treatment with the EcoRV digested genome that is packaged in virus particles, we observed that a band of app. 2,500 bp was missing (Supplementary Figure S3). We conclude that this fragment only appears when digesting the linear genome and could represent one end of the linear virus genome. Analytic digest of virus and host DNA with *Dpn*I, showed that both the host and the virus genome are dam methylated (Supplementary Figure S3).

**Figure 2:**
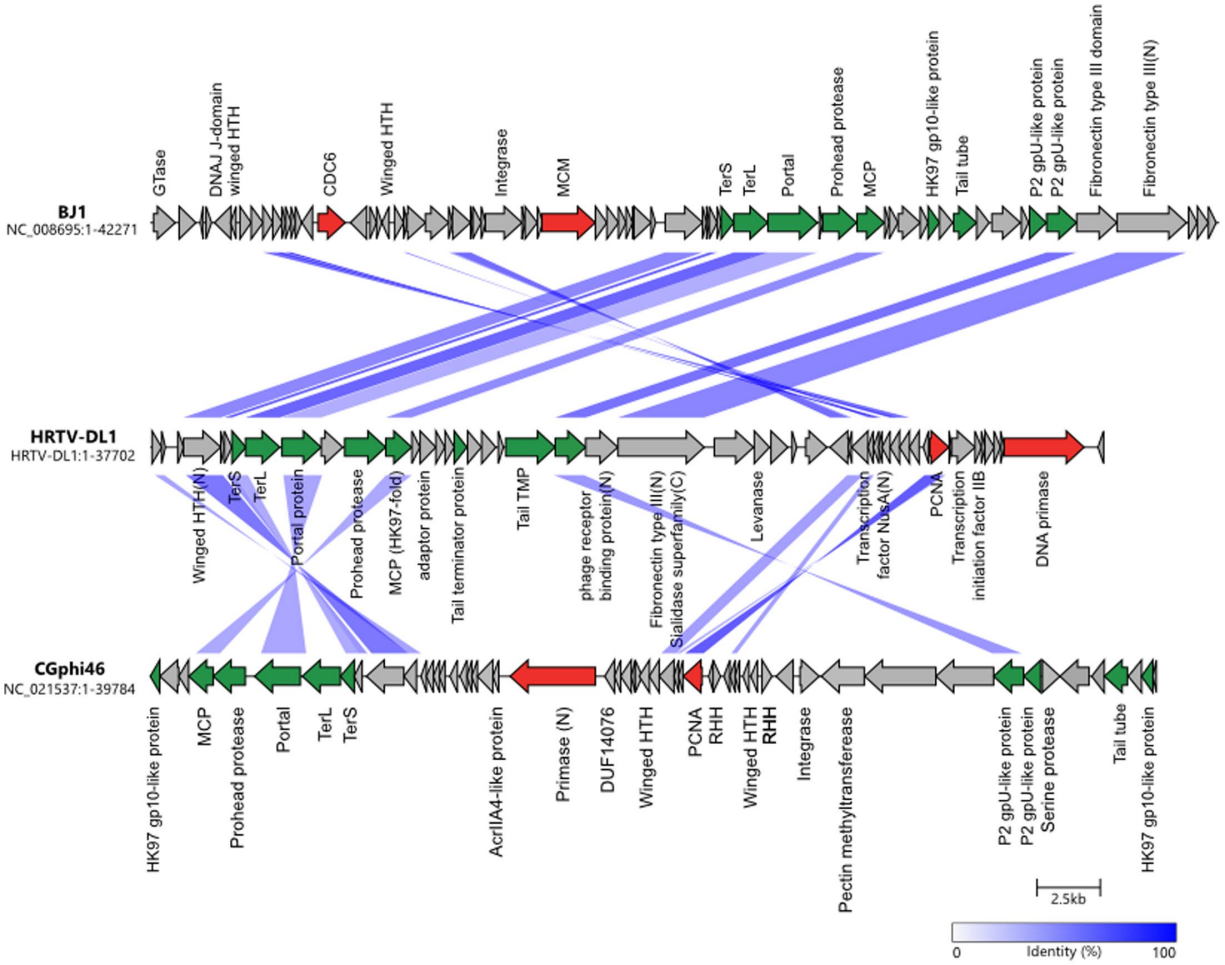
Comparisons of genomes HRTV-DL1 (OP630575) with BJ1 (NC_008695) and CGphi46 (NC_021537) belonging to the *Flexireviridae.* Protein functions are indicated above or below the corresponding ORFs. Genes encoding virus morphogenesis–related proteins are colored in green, whereas replication-related genes are colored in red. Homologous genes shared between viruses are connected by shadings of different degrees of blue based on the amino acid sequence identity. Figure was generated with clinker & clustermap.js (Gilchrist and Chooi, 2021).

Sequencing and assembly of HRTV-DL1 genome revealed a single circular contig of 37.7 kb, with no indication in the sequence data for discrete ends. The genome of HRTV-DL1 contains 49 putative open reading frames (ORFs) (Figure 2 and Supplementary Table S1). Using Hidden Markov models (HMM) (Söding, 2004) and domain prediction (Blum et al., 2020) we can predict the function for the products of about 40% of these ORFs (Supplementary Table S1). Two genes were identified that we propose to be involved in virus genome replication, both are also encoded by arTV CGphi46 (Figure 2). ORF48 product is predicted as primase-helicase, probably generating a short primer that is then elongated by the host DNA polymerase. ORF41 product is predicted as DNA polymerase sliding clamp, binding the DNA polymerase and preventing its dissociation from the template DNA. Two genes could be involved in transcription. ORF43 product, a predicted transcription initiation factor IIB, might be responsible for recruiting the host RNA Polymerase and ORF32, with weak hits to antitermination protein NusA, could associate with the host RNA polymerase elongation complex. ORF29 product is a predicted adenine-specific methyltransferase and 100% identical to a host protein, probably recruited by the virus to protect its genome from host RM-systems. ORF7 and ORF8 products were identified as small and large subunit of the terminase, responsible for genome packaging into the virus particle.

Proteins building the virus particle were identified by mass spectrometry of purified virus particles and confirm our predictions (Supplementary Tables S1, S2). The major capsid protein exhibits the HK97 fold and is most similar to that of arTV BJ1 (45% identity). Additionally, we could identify the phage portal protein, two capsid proteins with similarity to *Natrialba* virus PhiCh1 capsid proteins, the putative head-tail adaptor, the tail tape measure protein, and the tail terminator protein. ORF18 and ORF25 products, identified in the virus particle, did not show any similarities to other viral proteins, therefore, their role within the virus particle remains enigmatic. Two proteins that could be involved in host attachment were identified. ORF24 product, that is present in virus particle, exhibits a immunglobulin-like fold (fibronectin type III domain) at the N-terminus. This domain is commonly found in bacterial phage tails and has been shown to exhibit membrane binding activity (Fraser et al., 2006; Kageyama et al., 2009; Pell et al., 2010). In the C-terminus we identified a sialidase domain. Sialidase (neuraminidase) is known to cleave glycosidic linkages and is essential for some eukaryotic viruses (von Itzstein, 2007). It could be involved in destabilizing the glycosylated S-layer and facilitating access to the host membrane. ORF23 product, also detected in virus preparations, shows similarities to a phage receptor binding protein with a high probability (*p* = 99.06). ORF4 product, with significant similarities to DNA double-strand break repair protein MRE11, and ORF43 product, annotated as transcription initiation factor, were also detected in virus particles. Both could be associated with the virus genome. ORF4 product might facilitate circularization of the virus genome within the host, while ORF43 product could recruit the host RNA polymerase ensuring timely transcription of the viral genome after injection.

Genome comparison with 63 arTV (Liu Y. et al., 2021) and phylogenetic tree construction (Supplementary Figure S4), suggests HRTV-DL1 to be closest related to arTV BJ1 (Pagaling et al., 2007) and CGphi46 (NC_021537) (Figure 2) that have been classified into the *Flexireviridae* family. However, HRTV-DL1 only shares nine genes with CGphi46, but none with a sequence identity above 60%, and shares sixteen genes with BJ1 of which only three have a sequence identity above 60% (ORF6, ORF33, ORF34). Therefore, HRTV-DL1 should be classified into a different genus, that we propose to name *Deelavirus* in accordance with its origin (Deep Lake).

### Life cycle of HRTV-DL1

**Figure 3:**
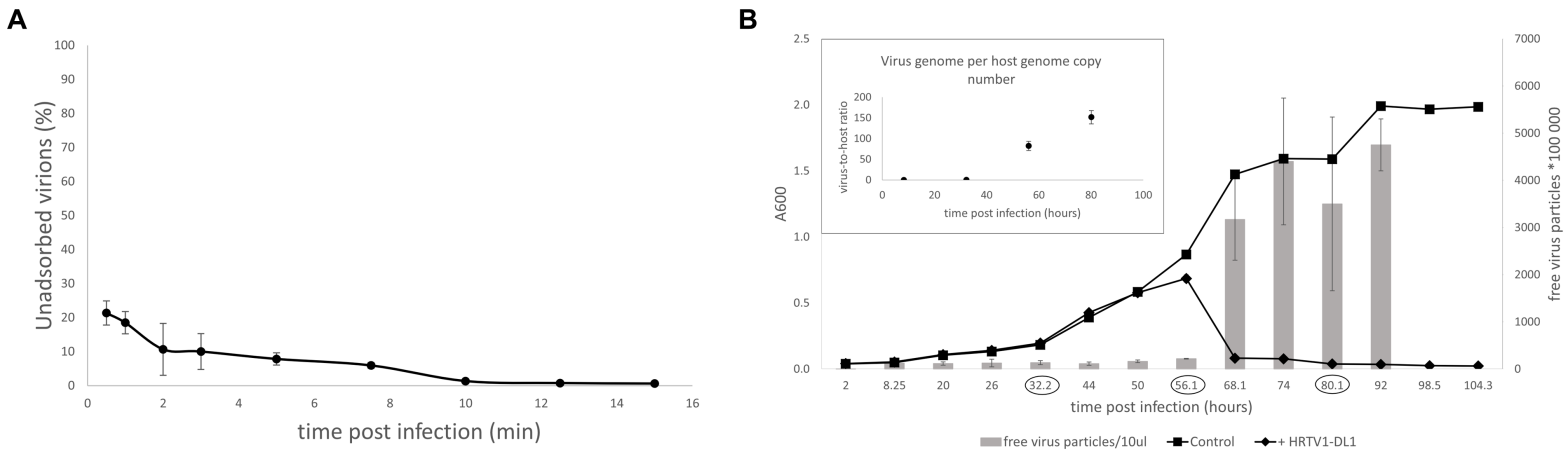
Life cycle of HRTV-DL1. **(A)** Adsorption of HRTV-DL1 to cells of *Hrr. lacusprofundi ACAM34_UNSW*. Cells were incubated with HRTV-DL1 at room temperature (20°C). The number of unbound virus particles was determined at different time points post infection by plaque assay. Graph represents one of three biological replicates. Error bars represent standard deviation from three independent experiments. **(B)** Growth curve of uninfected control and HRTV-DL1 infected *Hrr. lacusprofundi* ACAM34_UNSW. Free virus particles in 10ul culture supernatant were determined by plaque assay. Inset: Virus-host ratio determined by comparing copy numbers of host main chromosome and virus genome. Copy numbers were determined per ml cell culture by qPCR and samples were taken at time points encircled in the growth curve in the main figure. Graphs represent one of three biological replicates. Error bars represent standard deviation from three independent experiments.

To gain insights into the initial stages of HRTV-DL1 entry, we followed the adsorption of HRTV-DL1 to ACAM34 host cells. The adsorption was very rapid, with ∼75% of virions being bound to cells within the first 30 s of infection. Further adsorption plateaus after 15 min with 99% of particles bound to host cells (Figure 3A). Such a rapid adsorption is rather uncommon for haloarchaeal arTV. *Haloarcula hispanica* tailed virus 1 (HHTV-1), binds extremely slowly with virions adsorbed only after 3 h and binding of *Halorubrum* virus HRTV-1 was only detected after 25 min (Kukkaro and Bamford, 2009). Such diversity in adsorption rates indicates differences in adsorption mechanisms and receptors. The rapid adsorption of HRTV-DL1 suggests that the receptor is easily accessible and highly abundant on the cell surface. After successful infection, host cell lysis occurred between 56 and 68 h post infection (p.i.) at 28°C, which appears to be a long intracellular phase prior to lysis when compared to other haloarchael tailed viruses (Pietilä et al., 2013a). The virus-to-host ratio (VHR) was determined by qPCR as 95 viral genome copies per host main chromosome copy number a few hours prior lysis (Figure 3B). Interestingly, we observed changes to the cell morphology of infected *Hrr. lacusprofundi* at the onset of cell lysis. While the majority of the uninfected cells retained their rod-shaped morphology, infected cells tended to round up in the late stages of infection (Supplementary Figure S5). However, a significant increase in cell size, as shown for another archaeal virus (Liu J. et al., 2021), was not observed.

Both *Hrr. lacusprofundi* and HRTV-DL1 were isolated from a lake that experiences very low temperatures (Gibson, 1999). To determine whether low temperatures have an influence on the virus life cycle, we performed growth experiments at different temperature between 4°C and 30°C. Host growth rates change at different temperatures, consequently, lysis was observed at different time points post infection. However, lysis always occurred in early exponential phase at all temperatures (Supplementary Figure S6). Therefore, we conclude that temperature does not influence the life cycle of HRTV-DL1 under the laboratory growth conditions tested.

### HRTV-DL1 exhibits a narrow host range that uncovers genetic changes in the laboratory strain serving as host organisms

HRTV-DL1 was isolated from Deep Lake, that is dominated by 4 different haloarchaea (*Halohasta litchfieldiae* tADL, *Hrr. lacusprofundi*, halophilic archaeon DL31, and *Halobacterium* DL1) (DeMaere et al., 2013). CRISPR spacers against HRTV-DL1 were detected in the CRISPR loci of all except 1 species (*Halobacterium* DL1) (Supplementary Table S3). CRISPR spacers of all three different Deep Lake haloarchaea target different positions on the virus genome, indicating that they were acquired independently and not by gene transfer. Therefore, DL31 and tADL could be potential host organisms for HRTV-DL1. We tested all 4 species, but only *Hrr. lacusprofundi* ACAM34 was susceptible to infection. Adsorption assays revealed that attachment of the virus to all tested strains is impaired (Supplementary Figure S7), showing that infection is already compromised at the adsorption stage. We assume that the attachment site of HRTV-DL1 is highly diverse. Modifications of cell surface proteins, in particular the S-layer that was found to be highly distinct, has already been proposed as a virus exclusion mechanisms of Deep Lake haloarchaea (Tschitschko et al., 2015, 2018).

**Figure 4:**
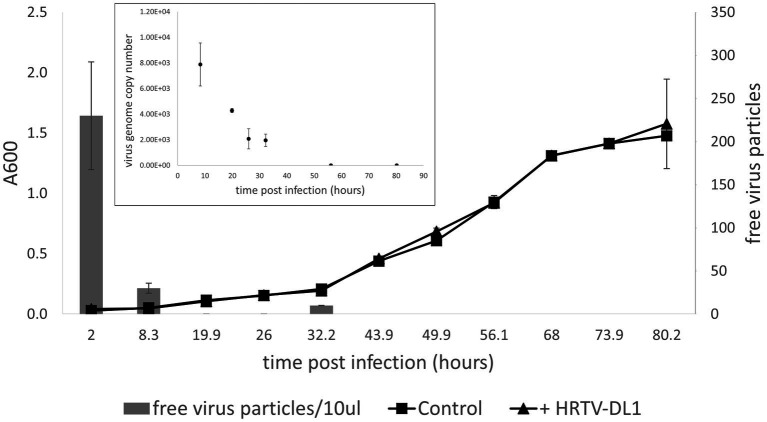
Infection of *Hrr. lacusprofundi* ACAM34_DSMZ with HRTV-DL1. Growth curve of uninfected control and HRTV-DL1 infected *Hrr. lacusprofundi* ACAM34_DSMZ. Free virus particles in 10ul culture supernatant were determined by plaque assay. Inset: Virus genome copy number determined per ml cell culture by qPCR. Samples were taken from the growth curve shown in the main figure. Samples in which virus genomes could not be detected by qPCR were set to 0. Graphs represent one of three biological replicates. Error bars represent standard deviation from three independent experiments.

When continuing characterization of HRTV-DL1 in a new laboratory, we used a fresh stock of *Hrr. lacusprofundi* ACAM34 provided by the DSMZ. Surprisingly, while the virus successfully adsorbed to the host cell and injected its genome, HRTV-DL1 was not able to complete a lytic life cycle in the DSMZ strain. No lysis was observed in cultures and virus DNA could not be detected within the host at 56 h p.i. (Figure 4). We subsequently assumed that the HRTV-DL1 susceptible strain, from now on referred to as ACAM34_UNSW (for University of New South Wales, Australia, the location of the laboratory the strain originated) had experienced genomic changes while being grown over a long time in the laboratory.

### Genome comparison of the ACAM34 laboratory strain with the ACAM34 strain from a culture collection reveals the loss of the majority of virus exclusion mechanisms in the laboratory strain

To determine genetic differences between the DSMZ strain (ACAM34_DSMZ) and the HRTV-DL1 sensitive strain (ACAM34_UNSW), we re-sequenced the genome of both strains (genomes provided in Supplementary Material).

ACAM34_DSMZ assembled into 4 contigs. Contig 1 is circular with a size of 2,735,247 bp, (coverage 1,080) representing the main chromosome (CHR1) with 99.99% identity to the published genome. Contig 2 is circular with a size of 525,899 bp (coverage 1,167), representing the secondary chromosome (CHR2) with 99.99% identity to the published sequence. Contig 3 is circular with a size of 431,344 bp (coverage 1,137), representing the plasmid pHLAC01 with 99.99% identity to the published sequence. Contig 4 is linear with the size of 6,176 bp matching a short region on CHR2 covering Hlac_3232 to Hlac_3234 with an insertion of a transposon within Hlac_3234. This contig has a lower coverage (432) than contig 2 and likely represents a variant of this region in the CH2 of the strain. In conclusion, the genome of ACAM34_DSMZ is to 99.99% identical with the published sequence.

**Figure 5:**
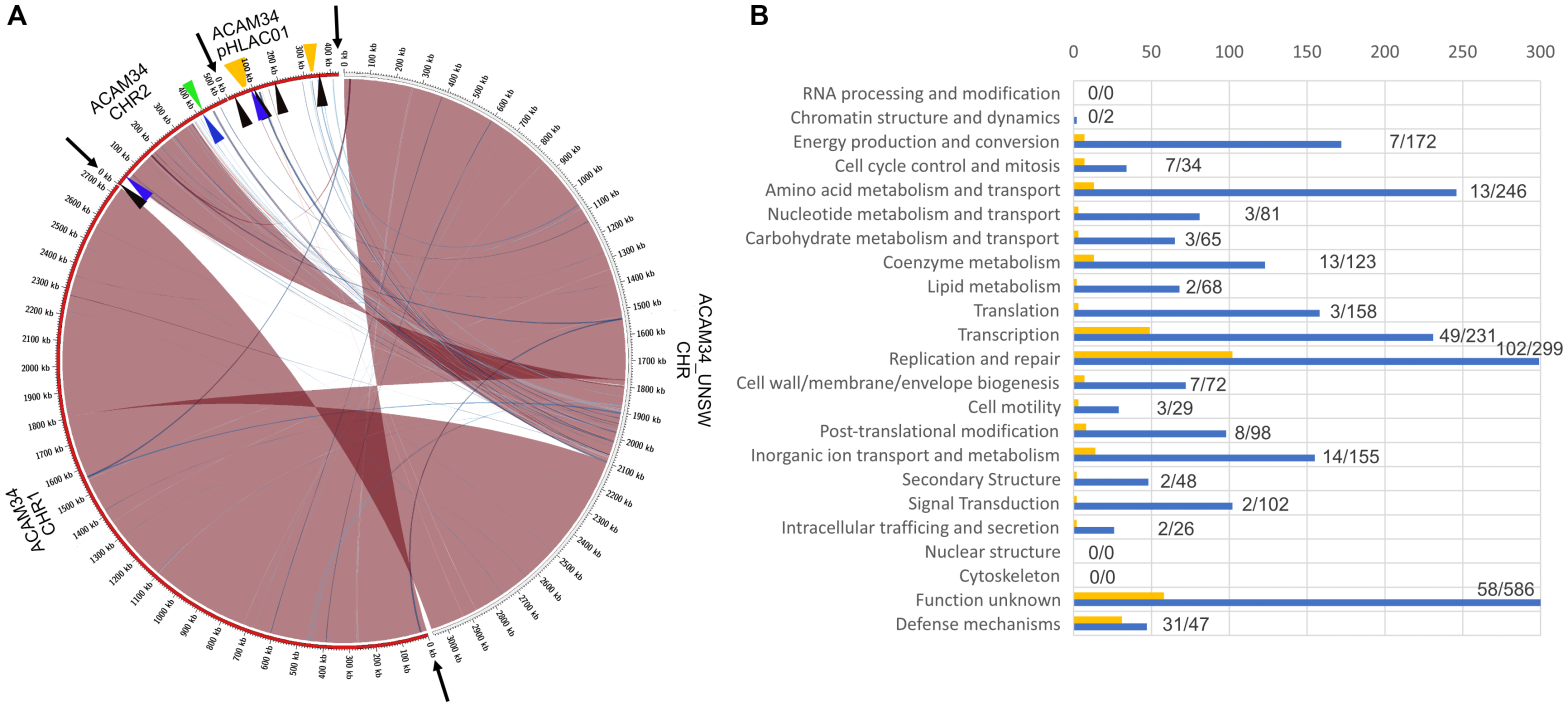
**(A)** Genome comparison of ACAM34_UNSW with the three replicons CHR1, CHR2 and pHLAC01 (NC_012029.1, NC_012028.1 and NC_012030.1) of the reference genome from NCBI (ATCC 49239). Interconnecting lines highlight regions present in both genomes (red/blue colors indicates same/reverse orientation on the two genomes). The figure highlights that the ACAM34_UNSW genome (on the right side, white outer ring) consists of a single replicon comprising the full length primary replicon (NC_012029.1) and parts of one secondary replicon (NC_012028.1) of the reference genome while pHLAC01 (NC_012030.1) of the reference genome (on the right side, red outer ring) is lost. Genomic regions shared between the two genomes were identified using NUCmer (Kurtz et al., 2004) and visualized with Circos (Krzywinski et al., 2009) wrapped within the script mummer2circos.py (https://github.com/metagenlab/mummer2circos). Black arrows indicate the borders between the three replicons of the reference genome. Colored arrows mark the position of the virus defense systems predicted with PADLOC (Payne et al., 2022). Black: DNA modification system, Blue: Argonaut-like protein, Green: BREX system, Orange: CRISPR system. **(B)** Functional profile of genes lost in ACAM34_UNSW. Blue bars represent the number of genes assigned to each particular functional category of all genes present on the ACAM34_DSMZ genome (X). Yellow bars represent the number of genes assigned to each particular functional category of all genes lost in ACAM34_UNSW (Y). Numbers behind the bars indicated genes lost / total amount of genes assigned to the category (Y/X). Functional classification of the *Hrr. lacusprofundi* genomes was performed using the cluster of orthologous genes (COG) database.

Surprisingly, ACAM34_UNSW assembled into only one single circular contig of 3,058,421 bp. Genome comparison with the published genome (Figure 5A) revealed that the entire plasmid pHLAC01 and about 38% (173 kb) of CHR2 is not present, while the remaining 61.11% of CHR2 (Hlac_2782 – Hlac_3106) is integrated into CHR1 between Hlac_1757 and Hlac_1759, two copies of ISH3 family transposase ISHla1 (Siguier et al., 2006) with 100% sequence identity. Hlac_2781 and Hlac_3106 flanking the CHR2 region integrated into CHR1 are also ISHla1 transposases that are 100% identical to Hlac_1757 and Hlac_1759. We suggest that the integration of CHR2 occurred by homologs recombination between the copies of ISHla1 and at the same time caused the loss of the remaining CHR2. The insertion of a large genomic fragment into the main chromosome due to transposition activity has already been reported for a *Halobacterium salinarium* laboratory strain (Pfeiffer et al., 2008), and the insertion of a large plasmid into the main chromosome has been reported for *Haloferax volcanii* (Hawkins et al., 2013). Apart from the insertion of CHR2, CHR1 remains highly conserved with an identity of 99.999%. One minor change is found in an intergenic region between Hlac_0599 and Hlac_0600 that is missing a stretch of 23 nucleotides. The integrated remains of CHR2 have three significant changes. First, a transposase was inserted into Hlac_2793, a predicted ADP-ribosylglycohydrolase, and disabled the gene. Second, Hlac_2835, with a MarR-like HTH domain within the N-terminus, has several changes on amino acid level within the C-terminus. And third, one of the two 16 s ribosomal RNA genes has a number of nucleotide changes that make up 3% of the sequence.

It remains unknown how the plasmid pHLAC01 was lost in ACAM34_UNSW. Analysis of the functional potential of the genes lost in ACAM34_UNSW revealed that the majority of them belong to the functional categories “Defense” (31 out of 47) and “Replication and repair” (102 out of 299), that are likely essential under virus infection or other stress condition influencing genome stability (Figure 5B). However, when grown under defined laboratory conditions these genes do not seem to be essential and are therefore likely too costly to be maintained. pHLAC01 exhibits the only CRISPR locus on the ACAM34 genome. This CRISPR locus contains 10 spacers that give a 100% match to the HRTV-DL1 genome (Supplementary Table S3). We therefore assume, that the resistance of ACAM34_DSMZ against HRTV_DL1 infection could be caused by the intact CRISPR system on pHLAC01, that is lost in ACAM34_UNSW. Nevertheless, there are also a number of other putative virus defense systems encoded on pHLAC01 and the region of CHR2 that is lost in ACAM34_UNSW, such as restriction modification systems (RM-systems), Argonaut proteins and a BREX system [Figure 5 and Supplementary Table S4, determined with PADLOC (Payne et al., 2022)]. These could also cause ACAM34_DSMZ resistance to HRTV-DL1 infection. Only one putative defense mechanism, an Argonaut protein (ACAM34UNSW_01791, Hlac_2785) (Willkomm et al., 2018), is still present in ACAM34_UNSW, leaving the strain basically unprotected against viral infection without any known virus exclusion mechanism.

### HRTV-DL1 infection induces the re-mobilization of CHR2 in ACAM34_UNSW

We analyzed the host response to HRTV-DL1 infection by determining transcriptional changes at two time points 32 h p.i. (middle phase, after infection has been established, time point 1 = T1) and 56 h p.i. (late phase, immediately before lysis, time point 2 = T2) for both strains ACAM34_DSMZ and ACAM34_UNSW.

**Figure 6:**
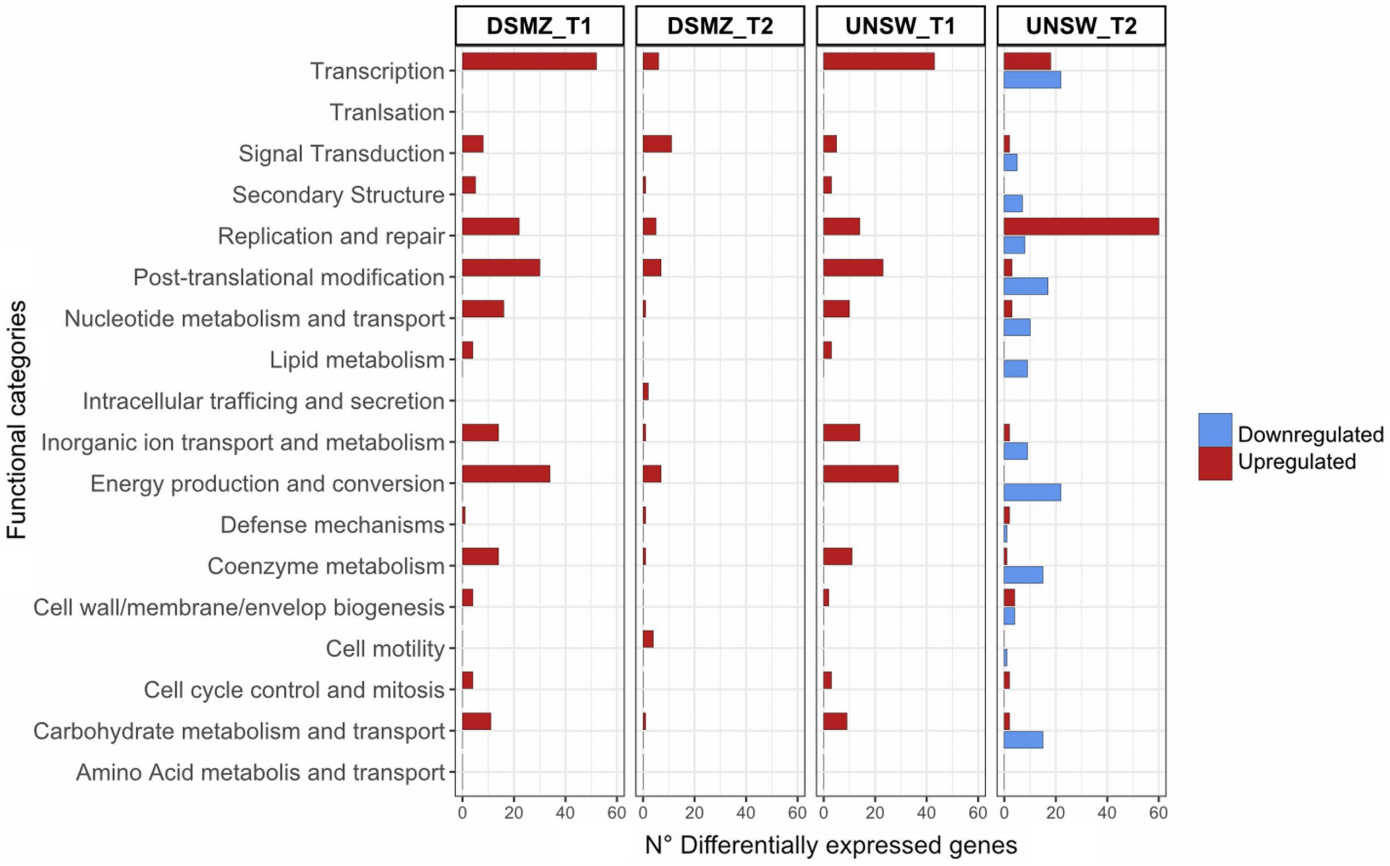
Functional profile of differentially expressed genes in ACAM34_DSMZ and ACAM34_UNSW under infection with HRTV-DL1. Bars represent the number of differentially expressed genes assigned to each particular functional category at a given time point (T1 = 32 h post infection, T2 = 56 h post infection). Functional classification of the *Hrr. lacusprofundi* genomes was performed using the cluster of orthologous genes (COG) database. The colors of the bars indicate if genes are upregulated (red) or downregulated (blue) respectively. Genes that were not assigned to any functional category are not displayed on the plot (for detailed information see Supplementary Tables S5, S6).

The ACAM34_DSMZ transcriptome recovered no reads for the virus genome in both samples, which is consistent with our results that the virus genome copy number is already very low at 32 h p.i. (time point 1 = T1) and not detectable at 56 h p.i. (time point 2 = T2) (Figure 4). None of the predicted virus defense mechanism (Supplementary Table S4), including the CRISPR system, showed significant upregulation (log2 > 1) (Supplementary Table S5). We therefore concluded that the time points chosen were too late post infection to determine the defense system active against HRTV-DL1 in ACAM34_UNSW. However, when comparing uninfected controls with infected samples, we still detect some changes to the host transcription. At T1 (32 h p.i.) 468 genes are upregulated more than 2 fold (log2 > 1) and none are downregulated, and at T2 (56 h p.i.) we detect 98 genes up- and 5 downregulated (Supplementary Table S5). When analyzing the functional potential of the upregulated genes at both time points, the majority falls into the categories transcription, replication and repair mechanisms, posttranslational modification, nucleotide metabolism and energy production and conversion (Figure 6).

The ACAM34_UNSW transcriptome recovered 0.2% of all mapped reads 32 h p.i. and 41% of all mapped reads at 56 h p.i. for the virus genome, consistent with the virus genome copy numbers increasing over time (Figure 3).

The transcriptional profile at time point 1 is very similar to that of T1 in ACAM34_DSMZ with 371 genes upregulated (log2 > 1) and none downregulated, and functional categories transcription, replication and repair mechanisms, posttranslational modification, nucleotide metabolism and energy production and conversion being the most upregulated (Figure 6). Additionally, we find the same genes among the 30 most upregulated genes, encoding for cold-shock proteins, ribosomal proteins, the ferredoxin Hlac_2176 and transcriptional regulators, being upregulated in ACAM34_UNSW and ACAM34_DSMZ, indicating that both strains exhibit a very similar response to HRTV-DL1 infection 32 h p.i. (Supplementary Table S6).

However, T2 shows a different profile, with 201 genes upregulated and 333 genes downregulated. The most upregulated functional category is ‘replication and repair’, consistent with a takeover of HRTV-DL1 and the degradation of the host genome (Supplementary Figure S8). Genes involved in metabolic processes seem to be subjected to downregulation. Amongst the 30 most upregulated genes, only two were also detected as upregulated in ACAM34_DSMZ 56 h p.i. (Hlac_0148, Hlac_0919). Surprisingly, the majority of genes on former CHR2 inserted in CHR1 (ACAM34UNSW_01788 to ACAM34UNSW_02100) was upregulated (Supplementary Table S6). Therefore, we assumed that this region might have been re-mobilized during virus infection and that the upregulation is due to an increased copy number of the secondary replicon. Indeed when comparing copy numbers of CHR1 with former CHR2 by qPCR in the same samples, we detect a slightly higher copy number for CHR2 in the late stage of infected samples (1.3x the copy number of the main chromosome) (Supplementary Figure S8). We conclude that CHR2 is mobilized only in a portion of cells in the population, because CHR2 usually has a copy number of 1.5–2 copies per CHR1 in ACAM34_DSMZ. When normalizing the expression values to the copy numbers, only 30 of 109 genes are still upregulated (log2 > 1), correcting the total number of upregulated genes from 201 to 122.

Within the 30 most downregulated genes at T2 we find genes encoding for ribosomal proteins, for proteins involved in amino acid metabolism, proteins involved in energy production and conversion and proteins involved in oxidative stress response (Supplementary Table S6), being a typical signature for a virus driven takeover of the host cell metabolism (Karlsson et al., 2005; Howard-Varona et al., 2020).

### The loss of virus defense mechanisms in ACAM34_UNSW leads to the activation of potential alternative virus exclusion mechanisms upon infection with HRTV-DL1

Only one gene, predicted to be potentially involved in virus defense (ACAM34UNSW_01791), an Argonaut related nuclease [PADLOC (Payne et al., 2022)] that is still present in ACAM34_UNSW, is slightly upregulated (log2 = 0.9) in ACAM34_UNSW, but not in ACAM34_DSMZ (Hlac_2785) at any time point. We suggest that ACAM34UNSW_01791 is activated in ACAM34_UNSW due to the lack of other defense mechanisms, however, its activity does not seem to interrupt the lytic cycle of HRTV-DL1. Whether it has an impact on the efficiency of infection or virus production remains to be determined. To identify other potential virus exclusion mechanisms we analyzed the 30 most upregulated genes. We propose that some of these genes might be implicated in virus exclusion of ACAM34_UNSW, that has lost all computational identifiable virus defense systems. The predicted functions of the upregulated genes are summarized in Supplementary Table S6 and some of particular interest, are discussed below.

ACAM34UNSW_01107 (Hlac_1086), shows significant similarities (HMM, Probability 99.95, E-value: 8.2e-26) to Transposon-associated TnpB, that is guided by an RNA derived from a sequence upstream of the TnpB (Karvelis et al., 2021) and also shows significant similarities (HMM, Probability 99.95, E-value: 8.2e-26) to ACAM34UNSW_01107. Interestingly, we do find a short (118 amino acids) ORF (ACAM34UNSW_01107a) upstream of ACAM34UNSW_01107 that is highly similar to the N-terminus of Hlac_2960, also showing high similarity to RNA-guided DNA endonuclease TnpB. ACAM34UNSW_01107a might represent TnpA that is also very small in size (140 amino acids) and responsible for the mobilization of TnpB. In absence of any other virus defense mechanism in ACAM34_UNSW, this gene might play an important role as a virus exclusion mechanism.

Two hypothetical proteins (ACAM34UNSW_01618 and ACAM34UNSW_01620) within the 30 most upregulated genes, enclose a predicted chromosome maintenance protein (SMC), ACM34UNSW_01619 (log2 = 1.1, T2). Additionally, another predicted SMC, ACM34UNSW_01796, is also slightly upregulated (log2 = 1.1, T2). SMC complexes were found to interfere with virus replication in humans (Gibson and Androphy, 2020; Han et al., 2022). Additionally, SMC-like proteins were found to be associated with the recently discovered Wadjet system, that has been shown to exclude circular foreign DNA in bacteria (Doron et al., 2018; Liu et al., 2022). Since both ACAM34UNSW_01618 and ACAM34UNSW_01620 do not show any similarity to other proteins involved virus defense, an antiviral activity of this gene cluster requires experimental verification.

Finally, another interesting candidate for a virus exclusion mechanism is ACAM34UNSW_02085 (Hlac_3088). The gene is a passenger gene of a transposon with transposase ACAM34UNSW_02084 (Hlac_3078), and could be mobilized similarly to other virus exclusion mechanisms (Broecker and Moelling, 2019) or antibiotic resistance genes (Babakhani and Oloomi, 2018). Hlac_3088 exhibits a PGF-TERM domain, an archaeal protein-sorting motif recognized by an archaeosortase (Abdul Halim et al., 2013), and is predicted to be a non-cytoplasmic protein with a TM domain. HMM search showed significant similarities with the S-layer protein of *H. volcanii,* however, Hlac_3088 is too small to be an S-layer protein. When comparing Hlac_3088 expression levels with those of the S-layer protein (Hlac_2976), Hlac_3088 has usually lower expression levels compared to the S-layer. However, at T2 it actually exceeds the expression levels of the S-layer gene (Supplementary Table S7). We suggest that Hlac_3088 is located at the surface of the cell and could protect the cell by hiding the receptor and interfering with the attachment of the virus, similar to TraT, encoded by the F plasmid of *Escherichia coli*, that interacts with OmpA and blocks phage adsorption (Achtman et al., 1977). Alternatively, it could stabilize the cell and prevent or at least delay cell lysis.

While none of the proposed mechanisms are fully effective against HRTV-DL1, because the virus is completing its lytic cycle, they might prevent the lysis of the entire population and allow virus escape mutants to evolve, and could be responsible for the long intracellular phase of the virus life cycle.

### HRTV-DL1 gene expression during infection reveals presence of a PATE with strong activity

Transcriptomic data from HRTV-DL1 infected ACAM34_UNSW cultures revealed that all annotated HRTV-DL1 ORFs were expressed, while ORF3 was, by far, the most transcribed ORF (Supplementary Table S8). When examining the reads mapping ORF3, we discovered that there is a peak that expands upstream of the annotated gene covering a region of approximately 300 bp and the majority of reads mapped antisense to the predicted coding sequence. Upon blasting the sequence against public databases, we discovered that it matched 99% (1 mismatch, 1 gap) to a region within the hosts genome and a region that was previously described in plasmid pR1SE infecting the same host (Erdmann et al., 2017). Further inspection revealed that this region represents a PATE (Palindrome-Associated Transposable Element), with a length of 489 bp that expands over the gap between ORF2 and ORF3. Only the C-terminal end of the PATE shows high expression, which includes the N-terminus of predicted ORF3, and expression occurs almost exclusively in reverse direction. We assume that ORF3 is a pseudogene and expression within this ORF is due to the PATE activity. In *Halobacterium salinarium* a PATE has been shown to be active as ncRNA (Gomes-Filho et al., 2015) and we imagine a similar function for this PATE. However, it is encoded by both the host and the virus, so it is difficult to predict whether its activity is beneficial for the host or the virus, and whether the virus acquired it from the host or vice versa.

### ACAM34_UNSW can develop resistance to HRTV-DL1 infection

To observe adaptation to HRTV-DL1 infection, we established long-term infected cultures, in triplicates, for ACAM34_UNSW with an MOI of only 1 to allow a recovery of infected cultures. For ACAM34_UNSW lysis was observed as expected between 45 and 70 h post infection and the cultures were maintained to allow the growth of HRTV-DL1 resistant clones. The cultures slowly recovered over a time frame of app. 200 h. After transfer of the recovered cells in fresh media, the cultures experienced another long lag phase of about 120 h before establishing a normal growth (Supplementary Figure S9). Cells from the recovered cultures were plated on agar plates to obtain single colonies and for each biological replicate we tested 24 colonies for infection with HRTV-DL1 by PCR. In 14 of the 72 tested colonies we could still detect the virus by PCR (Supplementary Figure S10), indicating that the majority of the cells were able to exclude the virus. We chose two escape mutants for further characterization.

### A large genomic deletion in escape mutant ACAM34_UNSW_2.14 identifies the S-layer as HRTV-DL1 receptor

**Figure 7:**
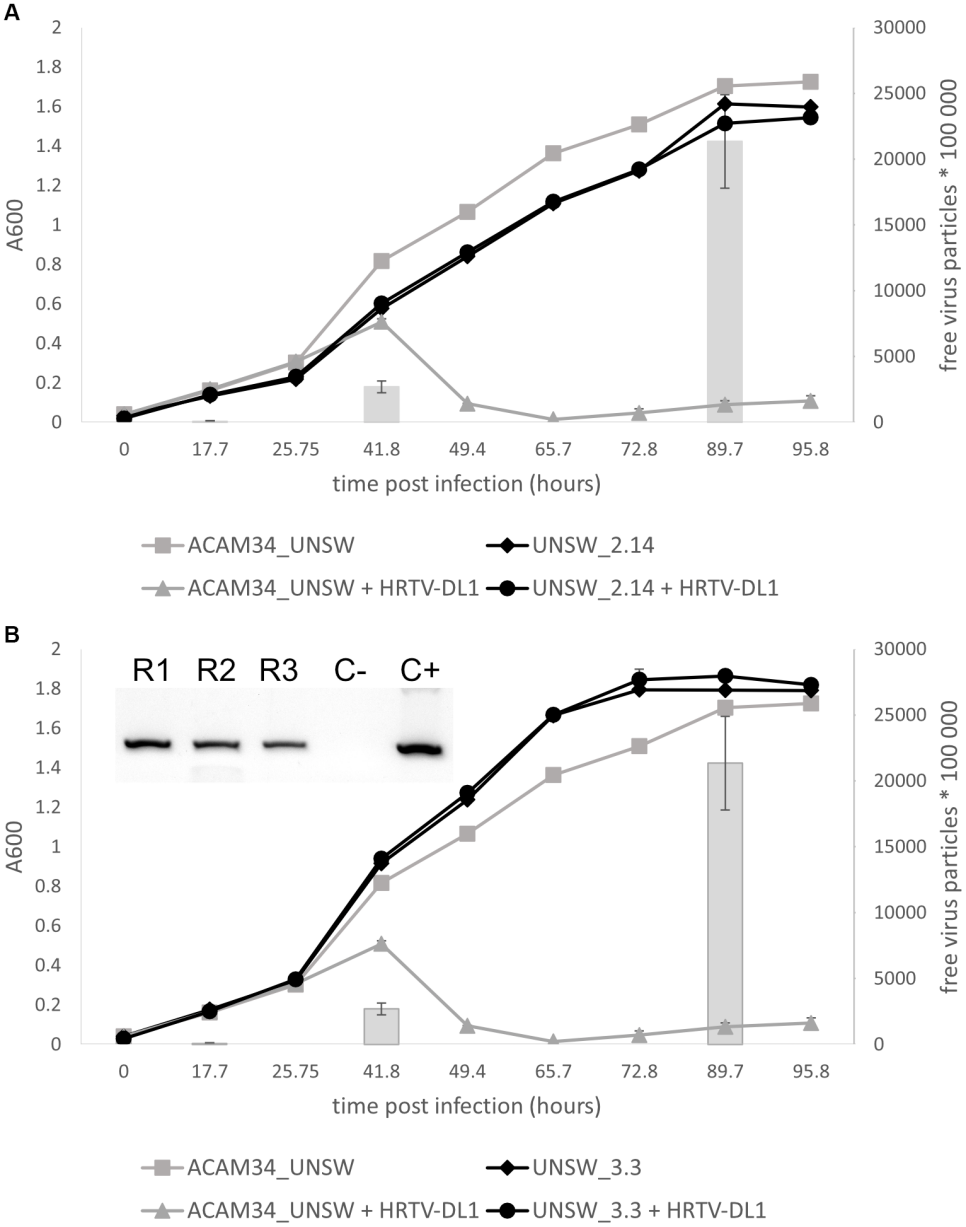
Life cycle of HRTV-DL1 in ACAM34_UNSW escape mutants. Growth curves of uninfected control and HRTV-DL1 infected *Hrr. lacusprofundi* ACAM34_UNSW, and escape mutants UNSW_2.14 **(A)** and UNSW_3.3 **(B)**. Free virus particles in 10 ul culture supernatant were determined by plaque assay and are presented by gray bars for the parental strain. For number of virus particles in supernatants of escape mutant cultures please refer to Supplementary Table S11. Graphs represent one replicate for the uninfected controls and one of three biological replicates for infected cultures. Error bars represent standard deviation from three independent experiments. Inset in **(B)** PCR confirming infection of three replicates of UNSW_3.3 with HRTV-DL1 (R1-R3 replicate 1–3; C− negative control; C+ positive control).

Escape mutant ACAM34_UNSW_2.14 was found to be fully resistant to HRTV-DL1 infection (Figure 7A). Infection is already impaired at the adsorption stage (Supplementary Figure S11), indicating that the receptor for HRTV-DL1 might have undergone changes in this mutant. Genome analyzes of UNSW_2.14 revealed a large gap covering the entire integrated CHR2 with two interruptions, indicating a massive rearrangement in several independent events. The region between ACAM34_UNSW_01838 (Hlac_2826) and UNSW_01867 (Hlac_2855), both transposases, is still present, as well as the region between the two transposases UNSW_01829 (Hlac_2819) and UNSW_01802 (transposase inserted into Hlac_2793). The coverage of these two regions is identical with the remaining chromosome, indicating that they are still integrated. We detect a few other changes, including twelve SNPs in intergenic regions, two silent SNPs in ORFs, three SNPs that lead to an amino acid substitution (Supplementary Table S9). None of these changes affect genes that would have an influence on virus-host interactions. However, the large deletion includes one of two S-layer genes (Hlac_2976) encoded by *Hrr. lacusprofundi*. Additionally, we also detected a deletion of two amino acids (D45 and S46) in the N-terminus of the second S-layer gene (Hlac_0412, 41% identity with Hlac_2976). Since adsorption of HRTV-DL1 to UNSW_2.14 is fully abolished, we conclude that the S-layer represents the receptor for HRTV-DL1, as it has been proposed for another arTV (HFTV1) (Schwarzer et al., 2023). Variability of S-layer genes of ACAM34 was previously detected by two ‘omics’-based studies of Deep Lake, and was suggested to be driven by an arms race between viruses and host (Tschitschko et al., 2015, 2018). Our results strongly support this hypothesis. We also propose that the deletion of Hlac_2976 causes the slightly reduced growth rate of UNSW_2.14 (Figure 7A). Cell morphology, as observed by light microscopy, does not seem to be significantly impacted (Supplementary Figure S12), suggesting that Hlac_0421 alone is able to maintain the S-layer.

### Escape mutant ACAM34_UNSW_3.3 confirms the S-layer as HRTV-DL1 receptor and suggest a Cdc6 protein to be involved in virus defense

Adsorption of HRTV-DL1 to escape mutant ACAM34_UNSW_3.3 is greatly reduced, but not fully impaired (Supplementary Figure S11). The virus genome could be detected in host cells, however, it is unclear whether it is actively replicated, and no cell lysis was observed and no virus particles were detected in culture supernatants (Figure 7B and Supplementary Table S11). We conclude that adsorption is partially impaired, and either virus genome replication, virus particle production or cell lysis is inhibited. Genome sequencing revealed 12 SNPs in intergenic regions, two silent SNPs, two aa substitutions observed previously in UNSW_2.14 and a frame shift in ACAM34UNSW_01960, a hypothetical protein. We do not expect that any of these mutations has an influence on the virus life cycle, since the majority of them are also present in UNSW_2.14 that has a different phenotype. The major change influencing virus-host interactions is an insertion of 12 aa (TPPTVSRLCFDT) between amino acids 209 and 210 of ACAM34UNSW_01982 (Hlac_2976), the S-layer gene that is also affected in UNSW_2.14. However, the mutation only accounts for 90% of the population, suggesting that the drastically reduced adsorption is caused by this mutation. Some virus particles can adsorb to the remaining 10% of the population, or can possibly attach to cells via the second S-layer protein (Hlac_0412). Hlac_0412 did not experience a mutation in UNSW_3.3, but we still see a dramatically reduced adsorption, indicating that either Hlac_2976 is the preferred receptor of HRTV-DL1, or the Hlac_2976 is preferably used by the cell to build the S-layer.

Despite the reduced infection efficiency, we detect virus genomes within cells, however, infected cells do not produce virus particles (Figure 7B). Only one other genomic change was detected in UNSW_3.3, the deletion of an origin of replication (ORI), that could possibly be responsible for preventing virus replication, virus particle production or cell lysis. ORIs are binding sites for Orc1/Cdc6 proteins and the deletion of an ORI could redirect Cdc6 causing the same effect as the upregulation of Cdc6 proteins. Indeed, the transcriptomics data show that while we only detected one (Hlac_3217) of fifteen annotated *orc1/cdc6* genes being upregulated in resistant ACAM34_DSMZ, five of seven remaining *orc1/cdc6* genes are differentially regulated in sensitive ACAM34_UNSW (Supplementary Table S5), including the *orc1/cdc6* gene adjacent to the destroyed origin (ACAM34UNSW_01845, Hlac_2833). Studies have shown that Cdc6 can bind dsDNA without sequence specificity (Feng et al., 2000), that dysregulation of Cdc6 expression can lead to inhibition of replication (Kundu et al., 2010) and that Cdc6 is also able to recruit the RNA polymerase I for rDNA transcription initiation (Huang et al., 2016). Additionally, a recent study showed that an Orc1/Cdc6 homolog, encoded by the archaeal virus SNJ2, is involved in regulating the lysogenic-lytic life cycle of SNJ2 (Chen et al., 2023). Indeed, we find an integrase encoded four genes upstream of the Cdc6 that is affected by the ORI deletion in ACAM34_UNSW, indicating that this Cdc6 might have been acquired from a virus genome. Therefore, we suggest that Cdc6 does affect the life cycle of HRTV-DL1, however, the mechanism needs to be investigated experimentally.

## Conclusion

In this work, we characterize a new head-tailed archaeal virus (HRTV-DL1) isolated from Deep Lake, Antarctica. HRTV-DL1 belongs to the *Flexireviridae* family, and we propose to classify HRTV-DL1 into the new genera *Deelavirus*. HRTV-DL1 exhibits a linear genome of 37.7 kb in size, that is replicated as circular genome within host cells, and is most likely terminally redundant similar to the genome of a number of other archaeal tailed viruses (Liu Y. et al., 2021).

HRTV-DL1 exhibits a lytic life cycle in a *Hrr. lacusprofundi* strain (ACAM34_UNSW) grown in the laboratory for a time that is not retraceable anymore. However, the type strain of *Hrr. lacusprofundi* obtained from the DSMZ did not show susceptibility to HRTV-DL1 infection. Genome sequencing uncovered that ACAM34_UNSW had undergone a massive genomic rearrangement and lost about 18% of its genome, a phenomenon that has also been described for other haloarchaea (Pfeiffer et al., 2008; Hawkins et al., 2013). While probably essential in the natural environment, this proportion of the genome, known to be highly variable in *Hrr. lacusprofundi* (Tschitschko et al., 2018), appears to be redundant when the strain is grown isolated and under laboratory conditions. Interestingly, the loss of genetic information included the majority of virus defense mechanisms present on the genome of the type strain, indicating that they could be very costly for the strain to maintain.

We were not able to determine with certainty the mechanisms that is responsible for HRTV-DL1 exclusion from ACAM34_DSMZ. However, the fact that ACAM34_UNSW lost the majority of virus defense mechanisms allowed us to uncover the receptor for virus binding and a number of undescribed putative virus exclusion mechanisms. Analysis of the HRTV-DL1 infected transcriptome of ACAM34_UNSW revealed the activity of potential TnpB-like RNA-guided DNA endonuclease, and a transposon passenger gene that could potentially prevent the virus from attaching to the host cell. Most interestingly, we discover the possible involvement of proteins shared between archaea and eukaryotes, a SMC-like protein and a CDC6-like protein, in virus defense, though antiviral activity of these proteins will require experimental verification. Infection of ACAM34_UNSW with HRTV-DL1 induces further rearrangements of the genome, in particular of the integrated CHR2, and allowed the isolation of escape mutants. We uncovered that one of two S-layer proteins is the preferred virus receptor, as recently also discovered for an arTV infecting *Haloferax gibbonsii* (Schwarzer et al., 2023), indicating that exhibiting two S-layer proteins could be a strategy of virus exclusion.

Our study provides important insights into the genome plasticity of *Hrr. lacusprofundi,* and highlights that genome plasticity has important implication for virus-host interactions. *Hrr. lacusprofundi* can be genetically modified (Gebhard et al., 2023), making *Hrr. lacusprofundi* and HRTV-DL1 to a great model system for studying virus-host interactions of archaeal tailed viruses. The new putative virus defense mechanisms discovered in this study can be investigated in detail, and possibly be identified in other organisms. ACAM34_UNSW itself can be used as model to study virus defense mechanisms. It is susceptible to a variety of viruses, including HRTV-DL1 and others (Nuttall and Dyall-Smith, 1993; Porter et al., 2005; Erdmann et al., 2017; Dyall-Smith, 2021; Alarcón-Schumacher et al., 2022), and individual virus defense mechanisms can be re-introduced to test their activity against diverse viruses.

## Materials and methods

### Isolation of HRTV-DL, strains and culture conditions

HRTV-DL, formerly described as DLHTHV (Tschitschko et al., 2018), was isolated from the supernatant of a colony that lysed upon propagation in liquid culture. Briefly, a sample, taken from Deep Lake (Antarctica) (Gibson, 1999) in 2014, was used to generate enrichment cultures in liquid medium (culture medium and conditions see below). Enrichment cultures were plated on solid media and a single colonie picked into liquid media. The colony was identified as *Halorubrum lacusprofundi* by 16 s rDNA sequencing using universal primers [21F and 1510R (Takahashi et al., 2014)]. After lysis was observed in the culture, cells and cell debris were removed by centrifugation (4,500 × *g*, 30 min), the supernatant of the culture (lysate) was filtered through 0.2 μm filters and used for infection of *Halorubrum lacusprofundi* ACAM34. *Halorubrum lacusprofundi* ACAM34 (DSM 5036), hereafter named ACAM34 _DSMZ, was obtained from the German Collection of Microorganisms and Cell Cultures (DSMZ). The parental strain of *Halorubrum lacusprofundi* ACAM34_UNSW was provided by either Peter Franzmann or John Bowman (not traceable anymore) and subsequently cultured in the laboratory (Ricardo Cavicchioli, School of Biotechnology and Biomolecular Sciences, The University of New South Wales, Sydney) from glycerol stocks stored at −80°C. The strain went through several rounds of culturing, plating and − 80°C storage in three different laboratories (UNSW and University of Technology, Sydney, Australia; Max Planck Institute for Marine Microbiology, Bremen, Germany) prior to re-sequencing. The number of generations that the strain had undergone during this time is not traceable. *Halohasta litchfieldiae* tADL (DSM 22187) was provided by the DSMZ. Halophilic archaeon DL31 and *Halobacterium* DL were isolated previously (DeMaere et al., 2013). All strains were grown in DBCM2 media (Dyall-Smith, 2021), with 5 g peptone and 1 g yeast extract added per liter. Incubation temperature was 28°C unless stated otherwise. Cultures were incubated in glass flasks aerobically at 120 rpm. Solid agar plates contained 16 g agar per liter and top layer agar contained 6 g agar per liter. Plaque assays were performed by mixing virus and host with 10 mL of top layer agar that was subsequently poured on solid plates and incubated at 28°C until growth was visible.

### Isolation and purification of HRTV-DL1 particles

For virus production, *Hrr. lacusprofundi* was grown in liquid culture to mid exponential phase, with an optical density at 600 nm (OD_600_) of 0.5. Cells were harvested (4,500 x *g* for 45 min), mixed with viral suspension with a multiplicity of infection (MOI) of 5–10 and incubated for 2 h at room temperature to allow viral adsorption. Treated cells were inoculated into 500 mL liquid cultures that were monitored by measuring optical density changes (OD_600_). After lysis occurred, cultures were centrifuged at 4,500 x *g* for 45 min to pellet the cells. The supernatant was recovered and viruses were subsequently precipitated with polyethylene glycol (PEG) 6,000 (10% w/v final concentration) and incubation at 4°C overnight. Then, viral preparations were collected by centrifugation (30,000 x *g*, 45 min, 4°C). Pellets were resuspended in DBCM2 salt solution (DBCM2 media without nutrient sources pyruvate, trypton and yeast extract), sterile filtered (pore size 0.2 μm) one time when used for plaque assay and twice when used in liquid culture infection assays. Virus solutions were stored at 4°C and were active for a minimum of 1 year. For downstream analyzes such as genome sequencing and mass spectrometry the virus particles were further purified by Cesium chloride (CsCl) density gradient centrifugation. The virus solution was treated with 200 U of DNase I and 50 μg/mL of RNase A to reduce host genomic contamination prior to loading on a CsCl density gradient (0.45 g CsCl /ml in DBCM2 salt solution) and centrifuged at 38,000 rpm for 22 h, 4°C (SW41 Ti Swinging-Bucket rotor, Beckman & Coulter). Bands containing virus particles were extracted with a syringe, diluted in three volumes of DBCM2 salt solution and re-precipitated with PEG (final concentration 10%, 4°C, overnight). After centrifugation at 30,000 x *g* for 30 min the resulting pellet was washed twice with DBCM2 salt solution and stored at −20°C before further processing.

### Imaging

For Transmission electron microscopy (TEM), virus containing solution was adsorbed for 5 min to carbon coated copper grids and stained for 1 min with 2% uranyl acetate (w/v in water). Electron micrographs were generated using JEM2100 Plus at 200 kV acceleration voltage. For light microcopy, cells were fixed with 1% glutaraldehyde for 1 h at room temperature and then stored at 4°C until imaging with a Zeiss AxioPhot microscope with AxioCam MRm.

### DNA extraction, manipulation and genome sequencing

Genomic DNA of all samples, if not otherwise stated, was extracted using genomic DNA extraction kit (Bioline, London, United Kingdom) according to the manufacturer’s instructions. Plasmid extraction was performed with ISOLATE II Plasmid Mini Kit (Bioline, London, United Kingdom) according to the manufacturer’s instructions. Nuclease digestions using specified restriction enzymes (New England Biolabs [NEB], 20 units) or exonuclease III (NEB, 5 units) were performed on 2–3 μg of DNA extracted from virus particles for 1 h at 37°C. PCR reactions were performed with the Q5^®^ High-Fidelity DNA Polymerase (NEB) and contained 0.02 units/μL of DNA polymerase, primer concentration of 0.1 μM for both forward and reverse, 1X of Q5 Reaction Buffer and 1X Q5 High GC Enhancer. The following program was used: 5 min at 95°C, followed by 35 cycles of 30 s at 95°C, 30 s at 68°C for annealing and 30 s at 72°C for elongation. Digested DNA and PCR products were separated on 1% agarose gels in Tris-borate-EDTA buffer and stained with SYBR^™^ Safe DNA stain (Invitrogen). For HRTV-DL1, UNSW_2.14 and UNSW_3.3 library preparation (FS DNA Library, NEBNext® Ultra™) and sequencing (Illumina HiSeq3000, 2 × 150 bp, 1 Gigabase per sample) was performed at the Max Planck-Genome-Center Cologne (Cologne, Germany). For PacBio sequencing of ACAM34_UNSW and ACAM34_DSMZ, DNA extraction, library preparation, sequencing (Pacific Biosciences Sequel, 4 samples on one SMRT cell) and assembly (‘Microbial Assembly’ function in PacBio SMRT^®^ Link v8.0 for ACAM34_DSMZ and flye assembly tool v2.6 (Kolmogorov et al., 2019) for ACAM34_UNSW was performed at the Max Planck-Genome-Center Cologne (Cologne, Germany).

### Genome assembly, annotation and phylogenetic analysis

After assembly with SPAdes (Bankevich et al., 2012), the HRTV-DL genome was manually closed to one contig using primers listed in Supplementary Table S10, followed by Sanger sequencing (MICROMON DNA Sequencing Facility, Monash University, Victoria, Australia) of PCR products. The HRTV-DL1 genome was assembled using metaviralSPAdes (Antipov et al., 2020). Genome annotation for all genomes was done using Prokka (Seemann, 2014) followed by manual corrections using conserved domain based searches (Blum et al., 2020) or hidden Markov model (HMM) based searches (Söding, 2004). Phylogenetic tree reconstructions from protein sequences was done with VICTOR under optimal settings (formula VICTOR d6), as implemented at the DSMZ webserver https://victor.dsmz.de. Prediction of termini was done using PhageTerm (Garneau et al., 2017).

### Protein content analysis

Protein content analysis of HRTV-DL1 particles was performed by mass spectrometry on purified virus pellets as described previously for plasmid vesicles and membrane vesicles (Erdmann et al., 2017). Peak lists were generated using Mascot Daemon/Mascot Distiller (Matrix Science, London, England) and initially submitted to the database search program Mascot (Matrix Science). Search parameters were: Precursor tolerance 4 ppm and product ion tolerances ±0.05 Da; Met(O) carboxyamidomethyl-Cys specified as variable modification; enzyme specificity was trypsin; 1 missed cleavage was possible; customized databases searched: *Hrr. lacusprofundi* ACAM34 and HRTV-DL. After genome sequencing of ACAM34_UNSW, results presented in Supplementary Table S2 were obtained using the SEQUEST search algorithm with Thermo Proteome Discoverer^™^ 2.5.0.400 (Thermo Fisher Scientific) with the same settings searching customized databases *Hrr. lacusprofundi* ACAM34_UNSW and HRTV-DL1.

### Virus infectivity and kinetics

To study the life cycle, cultures of *Hrr. lacusprofundi* (all strains) were synchronized using an adaptation of the “Stationary phase method” (Cutler and Evans, 1966). The strain was scratched from a  -80°C glycerol stock into liquid culture and grown up to an OD_600_ ~ 1. Then a 20-fold dilution step in fresh media was performed (final OD_600_ = 0.05) and cultures were then regrown up to OD_600_ ~ 1. Iterative dilution and growth of the culture were repeated twice before considering a culture synchronized. For infection with HRTV-DL1, cells from synchronized cultures (OD_600_ ~ 1) were collected by centrifugation, resuspended in 1 mL of fresh media and infected with HRTV-DL1 virus with a MOI of 5. After incubation (2 h, room temperature), cells were transferred into liquid cultures and growth was monitored by optical density (OD_600_). Long-term cultures were established by continuously diluting infected cultures at OD_600_ ~ 1 to OD_600_ ~ 0.05. Infection was confirmed by PCR using primers HRTV-DLF and HRTV-DLR (Supplementary Table S10). Viral titer in culture supernatants was quantified by plaque assay after removal of cells by centrifugation (11,000 × *g*, 10 min, room temperature [RT]). Intracellular virus titers and host chromosome copy numbers were quantified by qPCR. Briefly, samples of 2 mL culture in biological replicates were collected and pelleted (11,000 × *g*, 10 min, RT). Cell pellets were washed two times with 1 mL of fresh media and stored at −20°C upon DNA or RNA extraction. Quantification of *Hrr. lacusprofundi* and HRTV-DL1 genome copy number were carried out using a CFX96 Touch Real-Time PCR (Bio-Rad Laboratories, Inc., Hercules, CA, United States) and the software CFX Manager™ Software. Primers are listed in Supplementary Table S10. Each reaction (10 μL) contained 1X SsoAdvanced Universal SYBR^™^ Green Supermix (Bio-Rad) and primer concentrations as stated in Supplementary Table S10. The following amplification thermal cycling program was used for both primer sets: 5 min at 95°C, followed by 40 cycles of 30 s at 95°C and 30 s at annealing temperature stated in Supplementary Table S10, with readings taken between each cycle. Efficiencies of the assays were 95–102%, with R^2^ values ≥0.99 for all assays. The specificity of the qPCR was confirmed by unique signals in melting curves and gel electrophoresis of PCR products.

### Adsorption assays and host range assessment of HRTV-DL1

For adsorption assays, 5 mL of cells at OD_600_ = 0.5 were harvested by centrifugation (4,500 × *g*, 30 min, RT) and resuspended in 1 mL fresh medium. The cells were subsequently infected using a MOI of 5. At defined time intervals the adsorption was stopped by immediate centrifugation (10,000 × *g*, 5 min, RT). The number of remaining free virus particles in the supernatant was determined by plaque assay. A cell-free control (only media) served as control. Three different strains of haloarchaea [*Halohasta litchfieldiae* tADL, halophilic archaeon DL31, and *Halobacterium* DL1 (DeMaere et al., 2013)] were tested to determine the host range of HRTV-DL1. CRISPR matches to HRTV-DL1 were identified by searching for CRISPR loci using CRISPRs finder (Grissa et al., 2007) and blasting (BlastN) identified spacer against the HRTV-DL1 genome. Adsorption of HRTV-DL1 to the different strains was determined by adsorption assay as described above and the absence of HRTV-DL1 DNA within cells was confirmed by PCR on cell pellets 1 day and 5 days post infection.

### Transcriptomic analyzes

RNA extraction of frozen cell pellets with 3 biological replicates for the infected and 2 biological replicates for the controls, was performed with the Direct-zol™ RNA miniprep Kit (R2051, Zymo Research). RNA concentration and integrity were assessed using Nanodrop DS-11 Spectrophotometer (DeNovix) according to the manufacturer’s instructions. Library preparation and sequencing was done at the Max Planck-Genome-Center Cologne (Cologne, Germany). Briefly, ribosomal RNA were depleted prior to sequencing using the rRNA depletion Kit riboPOOL^™^, for *Haloferax volcanii* (88.36% identity to 16 s rDNA sequences of *Hrr. lacusprofundi*), siTOOLs Biotech^®^. Libraries were prepared with library kit NEBNext^®^ Ultra^™^ II RNA Library Prep Kit for Illumina and sequencing was performed on an Illumina HiSeq3000 sequencer, following a 1 × 150 run. Read trimming and mapping was performed with the “Map to reference” function (Mapper ‘Geneious RNA’) with medium-low sensitivity within Geneious Prime^®^ 2022.2.1. Expression values (FPKM values) were calculated using standard settings and comparison of expression levels were performed using DeSeq2 within Geneious Prime^®^ 2022.2.1 using default settings. Genes with *p*-values <0.01 and a fold change of at least two times (log2FC ≥ 1 or ≤ −1) were considered to be differentially expressed (DE).

### Analysis of ACAM34_UNSW escape mutants

For the isolation of ACAM34_UNSW HRTV-DL1 escape mutants we established long-term infected cultures as described above (*Virus infectivity and kinetics*), in triplicates. Cells were only infected with an MOI of ~1 to allow a recovery of infected cultures. After lysis of ACAM34_UNSW the cultures were maintained to allow the growth of HRTV-DL1 resistant clones. Cells from the recovered cultures were plated on agar plates to obtain single colonies. HRTV-DL1 infection was determined by PCR as described above. HRTV-DL1 life cycle in escape mutants and adsorption of HRTV-DL1 to escape mutants was determined as described above. Isolation of genomic DNA and genome sequencing is described above. For genomic analysis of escape mutants, reads were mapped against ACAM34_UNSW and HRTV-DL1 using ‘geneious mapper’ with medium-low sensitivity and default settings and variants were called using the Geneious function ‘Find Variations/SNPs’ with default settings in Geneious Prime® 2022.2.1. Variants with a coverage below 150 and a variant frequency below 75% were excluded from the analysis.

## Data availability statement

The datasets presented in this study can be found in online repositories. The names of the repository/repositories and accession number(s) can be found at: https://www.ncbi.nlm.nih.gov/, OP630574, https://www.ebi.ac.uk/ena, PRJEB56294, and https://www.ncbi.nlm.nih.gov/, PRJNA887929.

## Author contributions

CM: Investigation, Methodology, Writing – review & editing, Data curation. DT: Investigation, Writing – review & editing. LZ: Investigation, Data curation, Formal analysis, Methodology, Writing – review & editing. MR: Data curation, Writing – review & editing, Funding acquisition, Supervision. SE: Funding acquisition, Supervision, Writing – review & editing, Conceptualization, Investigation, Methodology, Visualization, Writing – original draft.

## Funding

The author(s) declare financial support was received for the research, authorship, and/or publication of this article. This work was supported by the Australian Research Council [DP170102576] Max Planck Society [Max Planck Research Group Archaeal Virology].

## Conflict of interest

The authors declare that the research was conducted in the absence of any commercial or financial relationships that could be construed as a potential conflict of interest.

The author(s) declared that they were an editorial board member of Frontiers, at the time of submission. This had no impact on the peer review process and the final decision.

## Publisher’s note

All claims expressed in this article are solely those of the authors and do not necessarily represent those of their affiliated organizations, or those of the publisher, the editors and the reviewers. Any product that may be evaluated in this article, or claim that may be made by its manufacturer, is not guaranteed or endorsed by the publisher.
