# Supplementary material for: Characterization of an archaeal virus-host system reveals massive genomic rearrangements in a laboratory strain
Source: Front Microbiol. 2023 Sep 18;14:1274068. doi: 10.3389/fmicb.2023.1274068 (PMC10544981; doi:10.3389/fmicb.2023.1274068)
Supplement: Supplementary file 1 [file Data_Sheet_1.zip › Supplementary figures and table captions.pdf]

Supplementary Information

Supplementary Figures

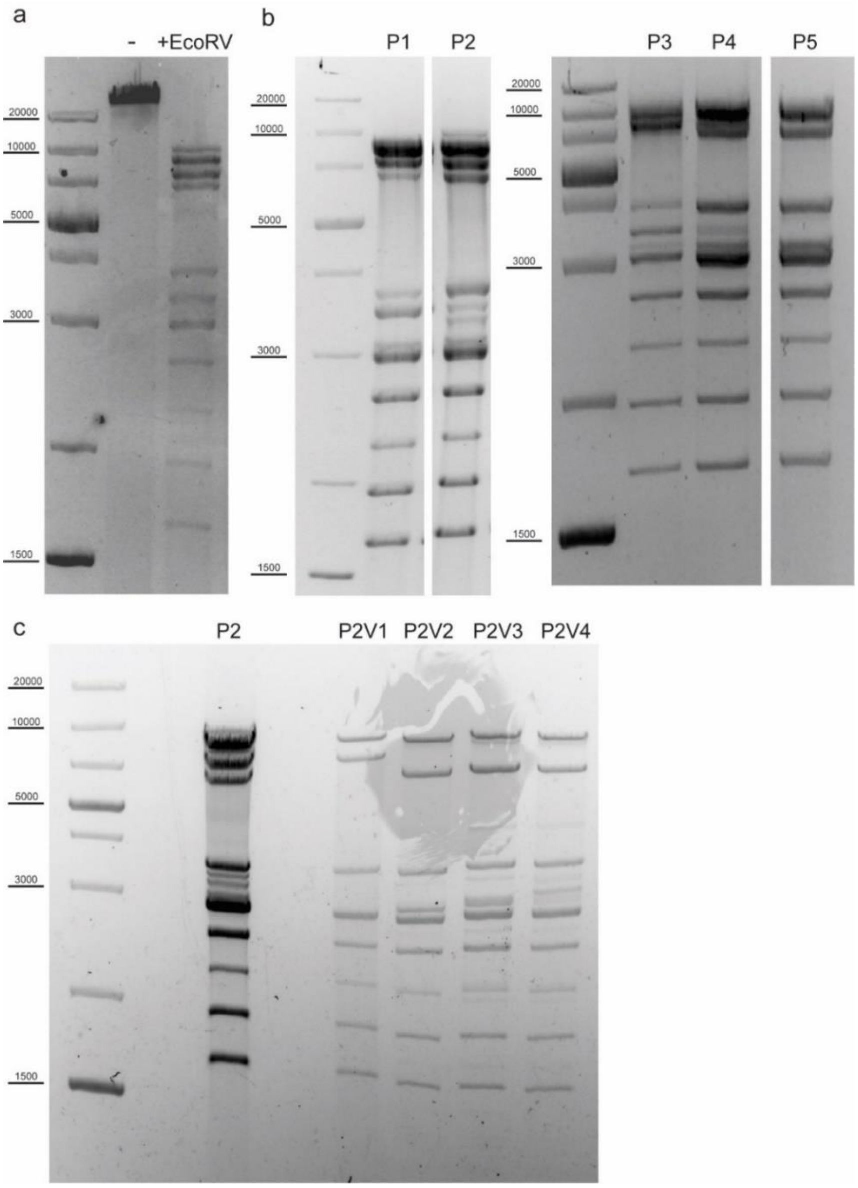

Figure S1. Analysis of genomic DNA of the original virus isolate and different variants isolated from single plaques. (**a**) Total DNA from virus particles isolated from the supernatant of a *Hrr. lacusprofundi* isolate [1] (HRTV-DL) undigested (-) and digested with *Eco*RV (+EcoRV). (**b**) EcoRV digest of DNA from HRTV-DL variants isolated from single plaques (P1-P5), formed after infection with the original virus preparation on *Hrr. lacusprofundi* ACAM34 lawns and propagated in the same strain. (**c**) EcoRV digest of DNA from HRTV-DL variants isolated from single plaques (P2V1-V4) formed after infection with P2 (same as in b) on *Hrr. lacusprofundi* ACAM34 lawns and propagated in the same strain. P2V1 (HRTV-DL1) was chosen for further studies. MW size marker is shown to the left of the gel (GeneRuler 1 kb Plus DNA Ladder, Thermo Fisher Scientific). DNA was separated on 1% agarose gels and stained with SYBR™ Safe DNA stain. Original gel images have been modified by excising separated lanes to improve visual presentation.

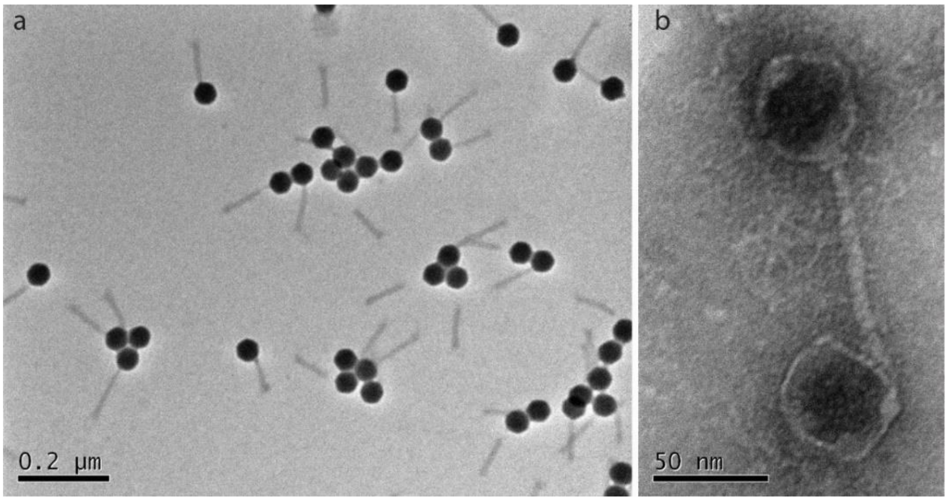

Figure S2. Transmission electron micrographs HRTV-DL1. (**a**) HRTV-DL1 virus particles purified from *Hrr. lacusprofundi* ACAM34 cultures. (**b**) Detailed view of HRTV-DL1 virus particle attached to a membrane vesicle or other virus particles. Samples were negatively stained with 2% uranyl acetate.

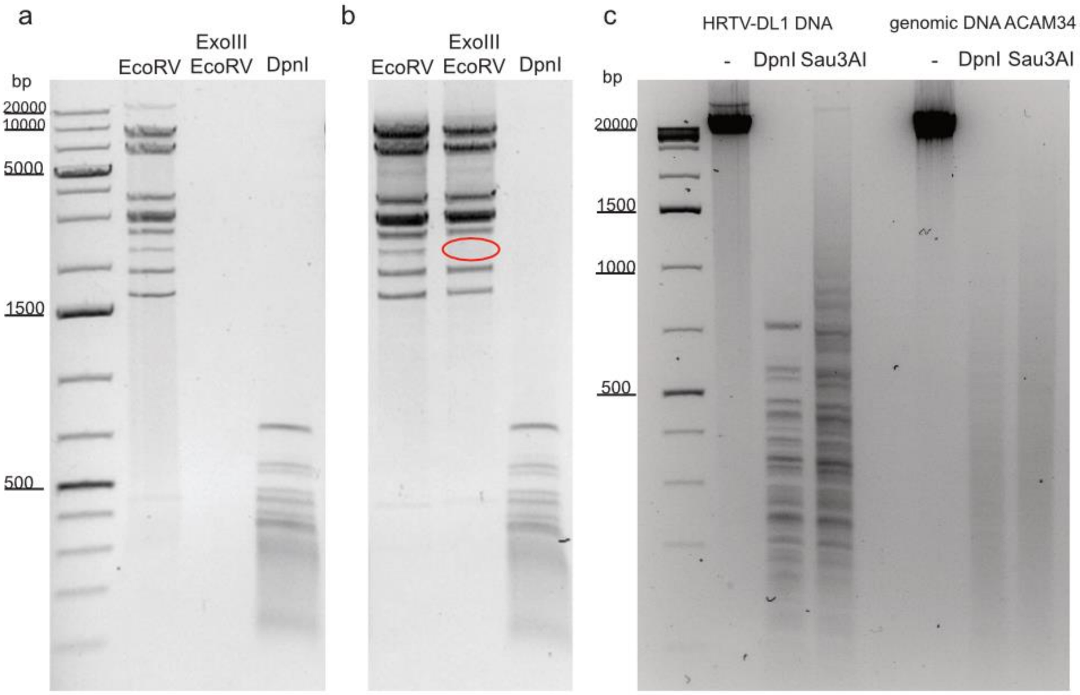

Figure S3. Analysis of genomic DNA of HRTV-DL1. (**a**) Total DNA of HRTV-DL1 isolated from purified virus particles digested with *Eco*RV, with Exonuclease III and subsequently *Eco*RV, and with *Dpn*I. (**b**) HRTV-DL1 DNA isolated from infected host cells (*Hrr. lacusprofundi* ACAM34) digested with *Eco*RV, with Exonuclease III and subsequently *Eco*RV, and with *Dpn*I. A band that is not present after Exonulcease III digest, is marked with a red circle. (**c**) Total DNA of HRTV-DL1 and *Hrr. lacusprofundi* ACAM34 undigested (-) and digested with *Dpn*I and *Sau*3AI. MW size marker is shown to the left of the gel (GeneRuler 1 kb Plus DNA Ladder, Thermo Fisher Scientific). DNA was separated on 1% (a,b) and 2% (c) agarose gels and stained with SYBR™ Safe DNA stain. Original gel images have been modified by cropping to improve visual presentation.

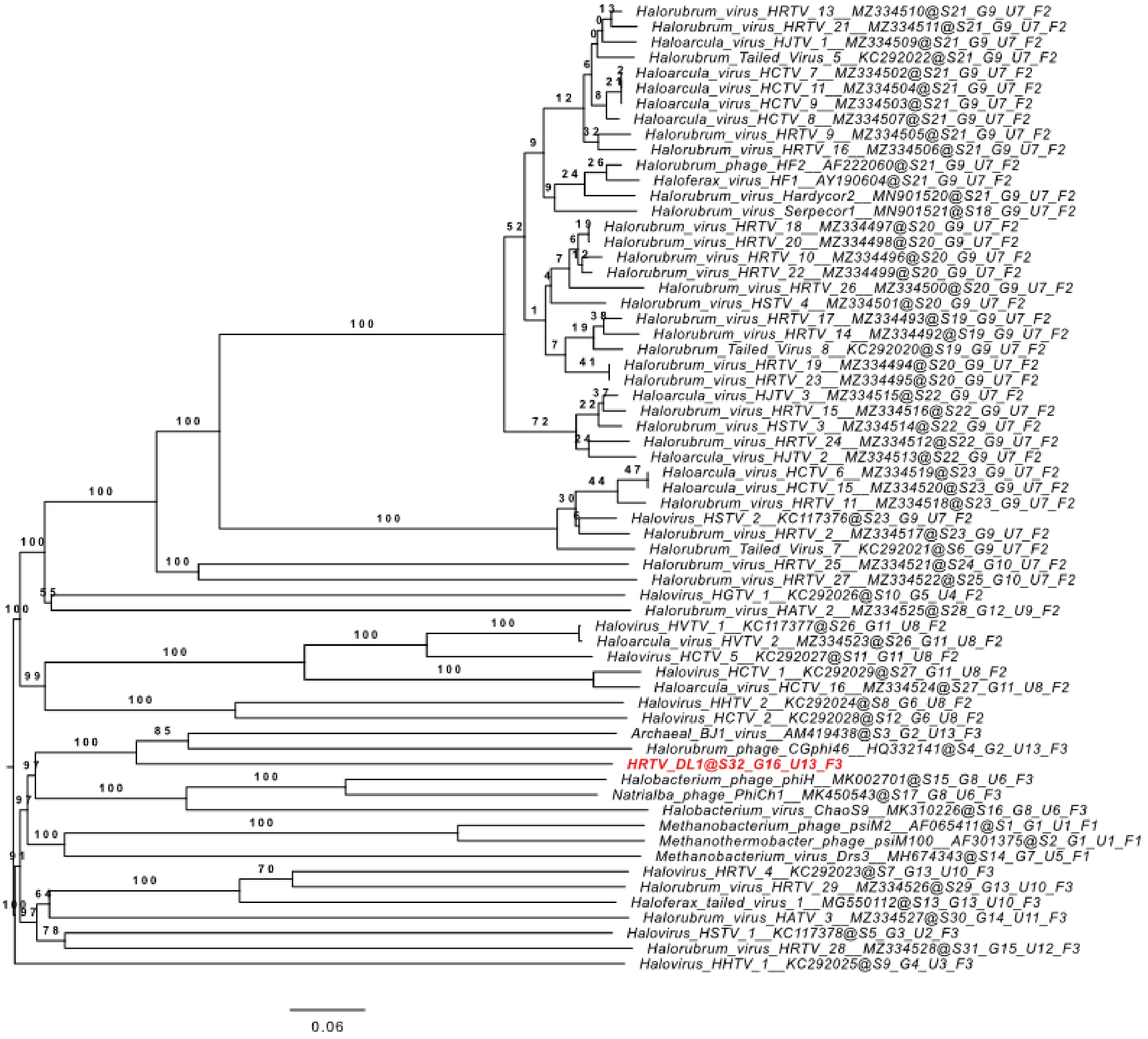

Figure S4. Phylogenetic tree reconstructions from protein sequences of 63 arTVs and HRTV-DL1 (highlighted in red). Phylogenomic GBDP trees inferred using the formulas d6 and yielding average support of 54 %. The numbers above branches are GBDP pseudo-bootstrap support values from 100 replications. The branch lengths of the resulting VICTOR trees are scaled in terms of the respective distance formula used.

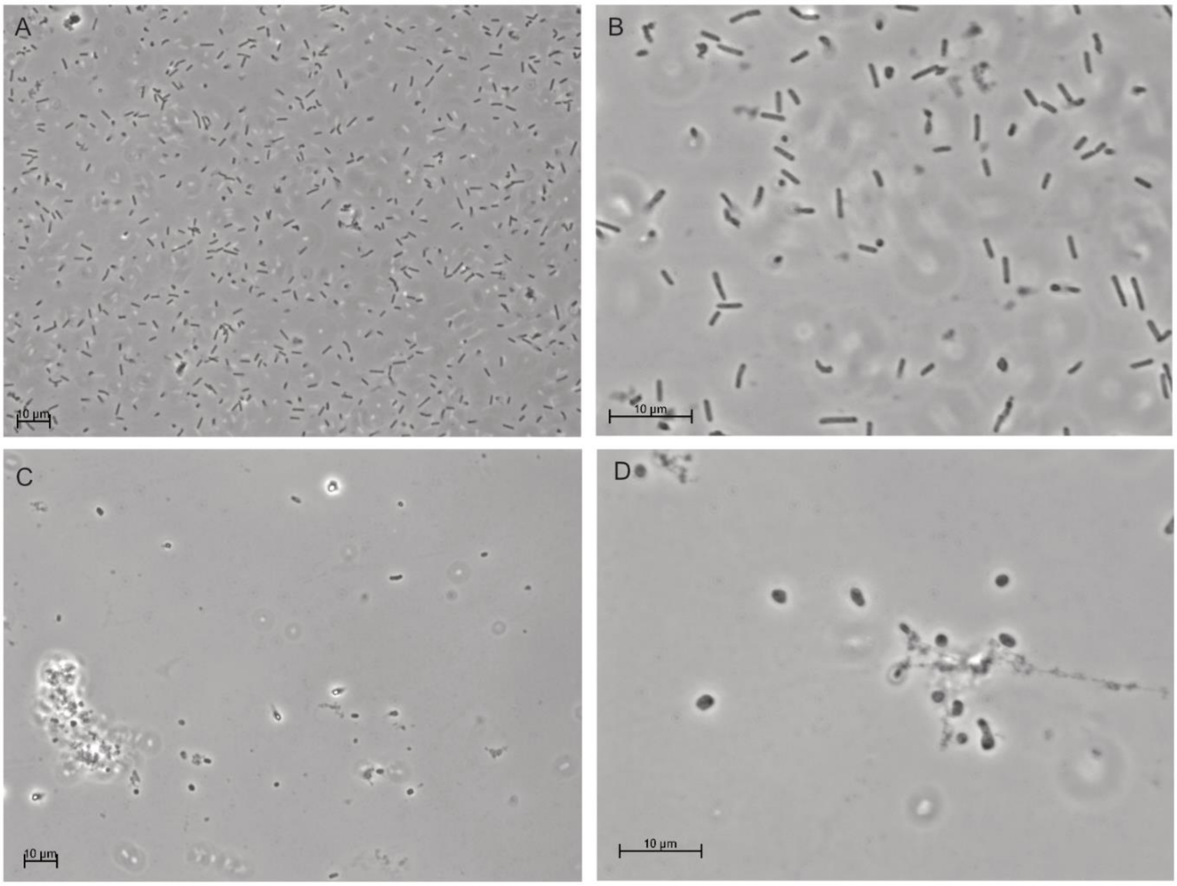

Figure S5. Cell-shape changes of HRTV-DL1 infected *Hrr. lacusprofundi* ACAM34_UNSW. Light Microscopy of uninfected (A and B) and HRTV-DL1 infected (C and D) *Hrr. lacusprofundi* 42 hours post infection at the onset of cell lysis. Cells are fixed with 1% glutaraldehyde. Pictures represent one of three biological replicates.

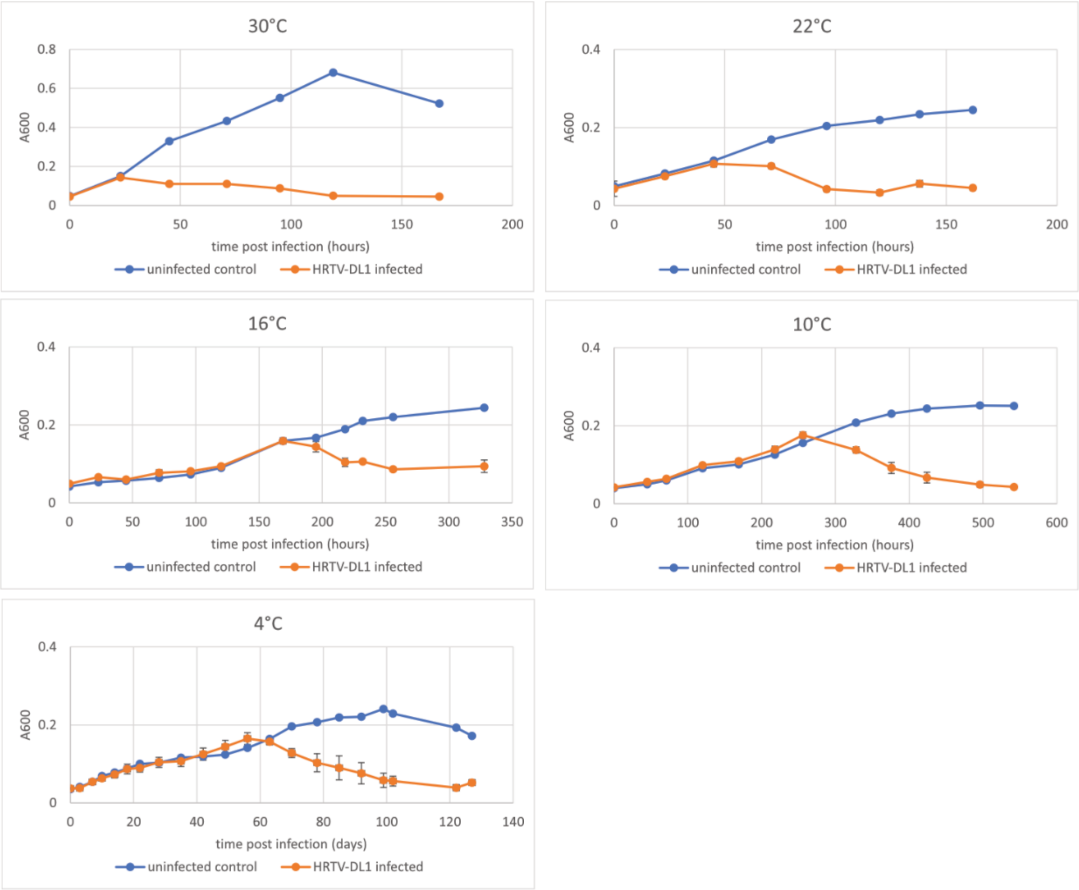

Figure S6. Virus life cycle at different temperatures. Growth curves of uninfected control and HRTV-DL1 infected *Hrr. lacusprofundi* ACAM34_UNSW at different temperatures. Graph 30 °C one replicate for control and infected. Graphs 22 °C, 16 °C, 10 °C and 4 °C with one replicate for the control and one representative of three biological replicates for the infected cultures. Error bars represent standard deviation from three independent experiments.

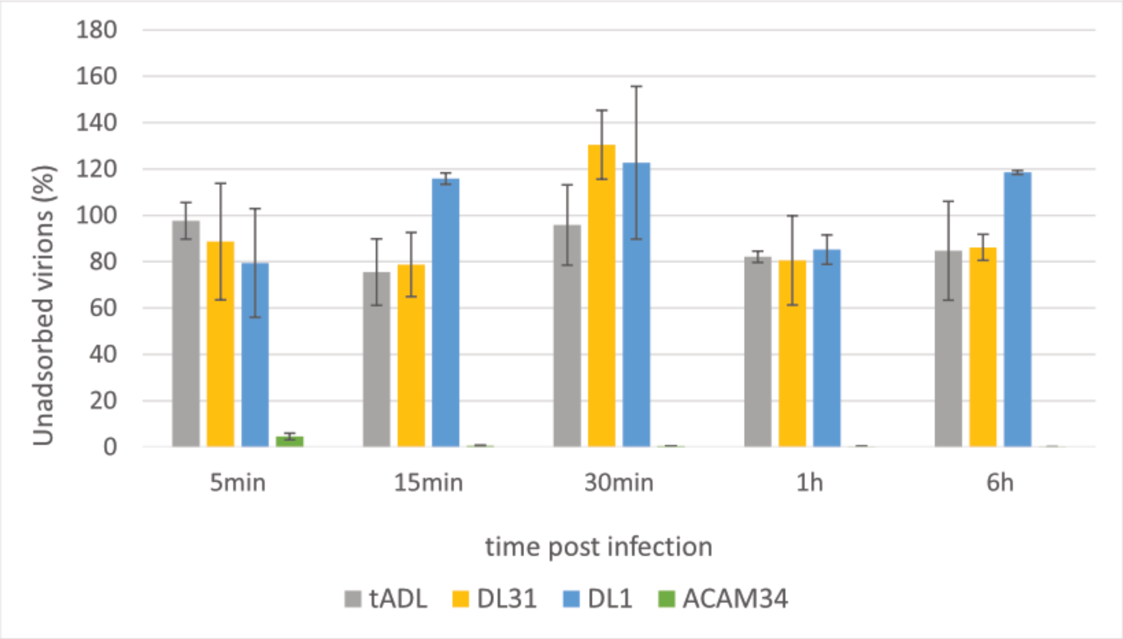

Figure S7. Adsorption assay of HRTV-DL1 with different potential host organisms. *Halohasta litchfieldiae* tADL, *Hrr. lacusprofundi* ACAM34_DSMZ, halophilic archaeon DL31, and *Halobacterium* DL1 were tested for HRTV-DL1 adsorption. Virus particles were incubated with host cells (or cell free media as control) for 5,15,30,60 and 360 min and number of not-adsorbed free virus particles in the supernatant was determined by plaque assay. Graphs represent one of three biological replicates. Error bars represent standard deviation from three independent experiments.

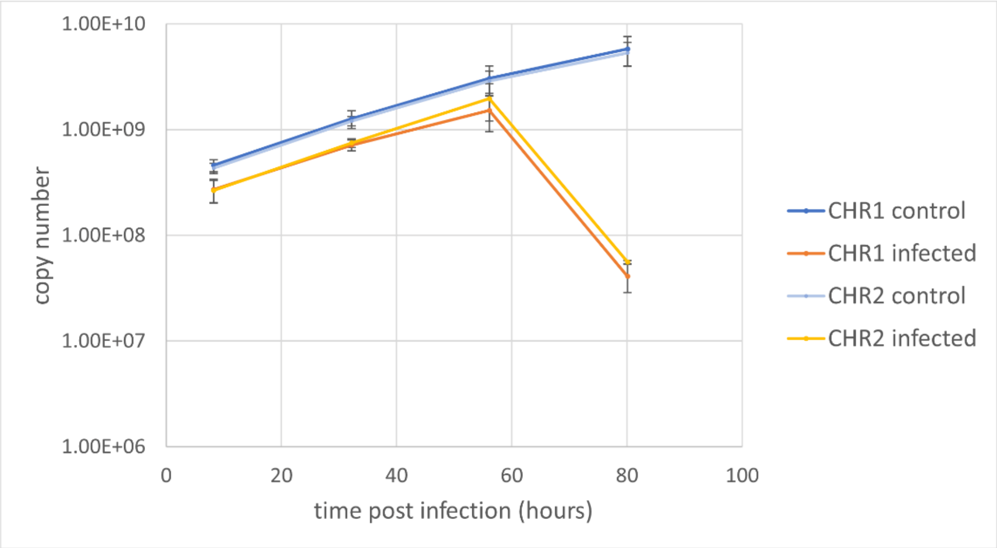

Figure S8. Copy numbers of ACAM34_UNSW main chromosome (CHR1) and the integrated secondary chromosome (CHR2) in uninfected controls and HRTV-DL1 infected cultures. Copy numbers were determined from 2ml cell culture by qPCR and samples were taken from the growth curve shown in Figure 5. Graphs shown are averages of three biological replicates. Error bars represent standard deviation from three independent experiments.

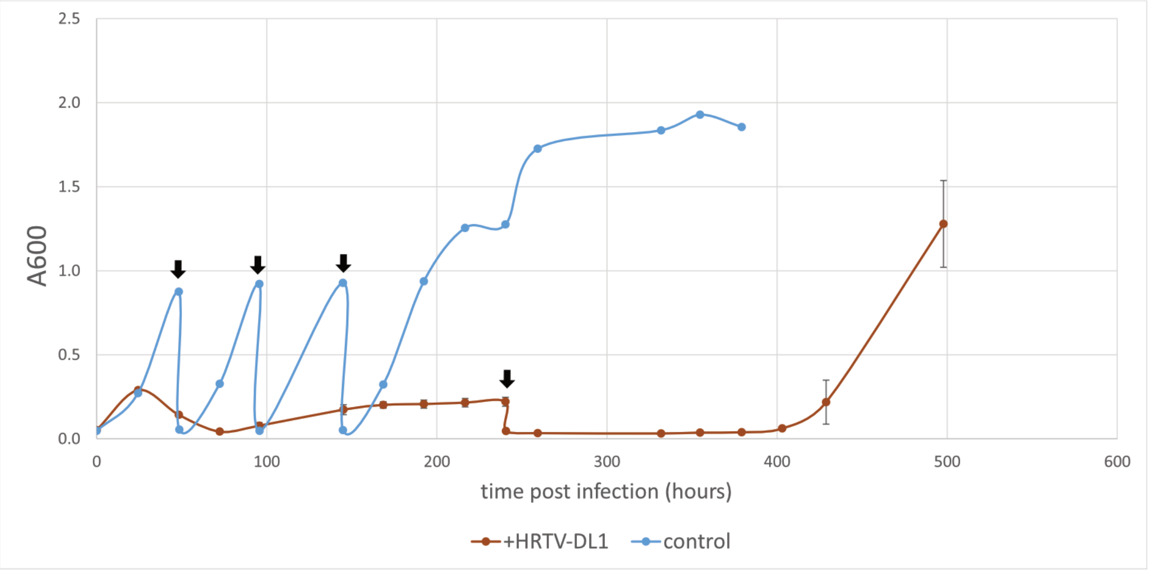

Figure S9. Long-term infection of *Hrr. lacusprofundi* ACAM34_UNSW with HRTV-DL1. Growth curve of uninfected control and HRTV-DL1 infected *Hrr*. *lacusprofundi* ACAM34_UNSW, with serial dilution of the culture in exponential growth as indicated by black arrows. One replicate for the control and one representative of three biological replicates for the infected cultures. Error bars represent standard deviation from three independent experiments (infected).

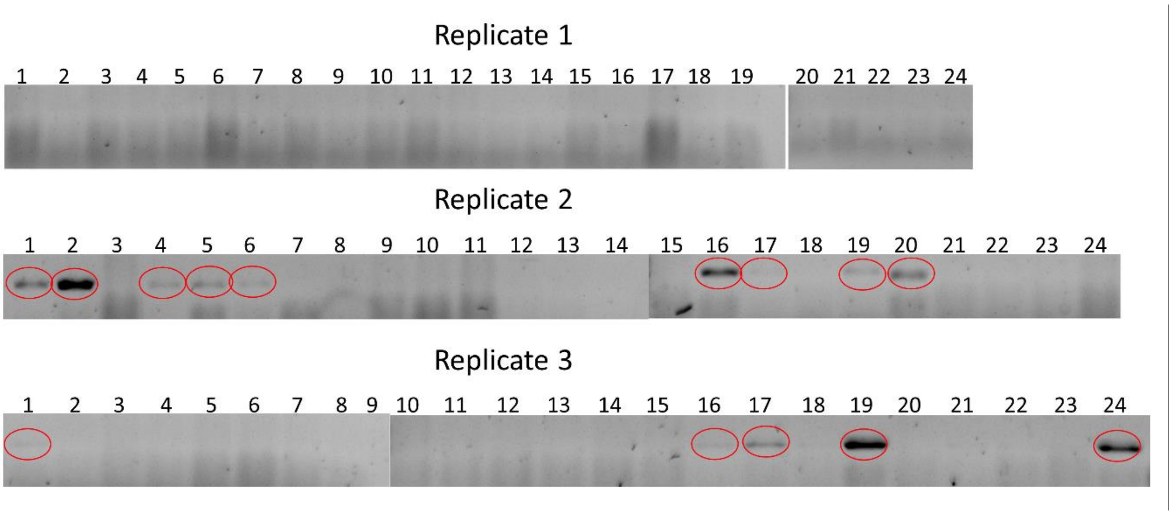

Figure S10. HRTV-DL1 in single colonies obtained from long-term infection of *Hrr. lacusprofundi* ACAM34_UNSW with HRTV-DL1. PCR on HRTV-DL1 in ACAM34_UNSW single clones obtained from three biological replicates of recovered long-term infected ACAM34_UNSW cultures. Colonies that showed a positive PCR signal for HRTV-DL1 are marked with red circles. Original gel images have been modified by excising separated lanes to improve visual presentation. DNA was separated on 1% agarose gels and stained SYBR™ safe DNA stain.

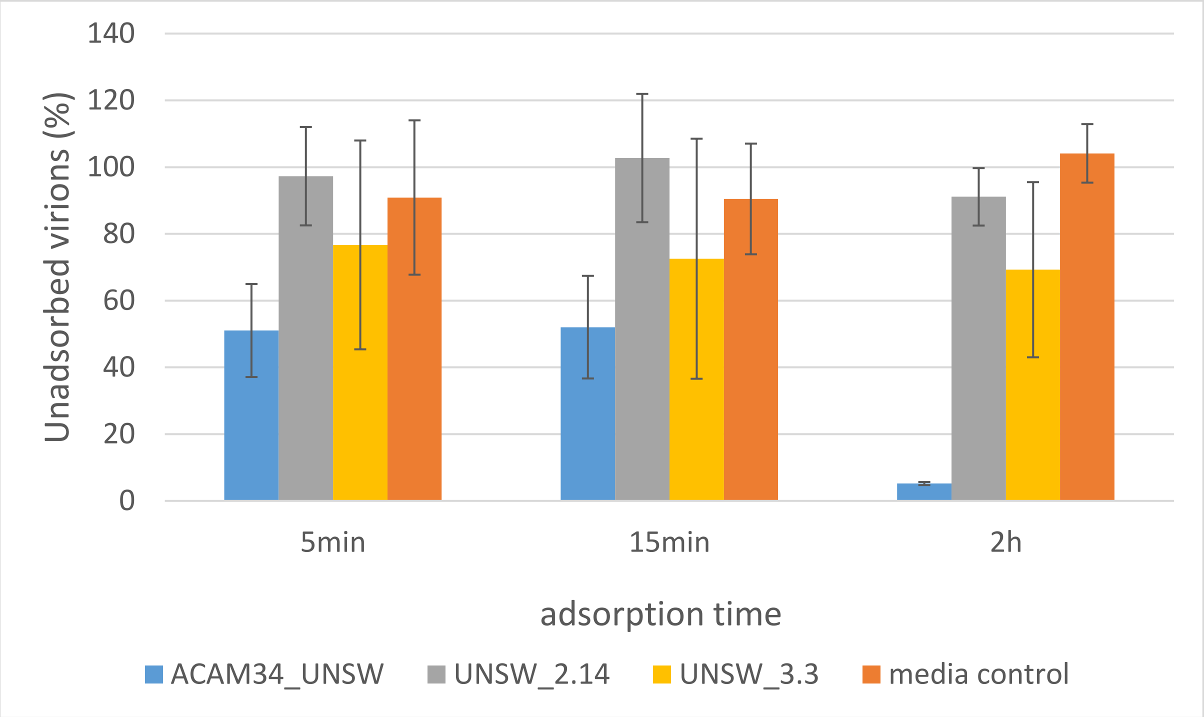

Figure S11. Adsorption assay of HRTV-DL1 with strains recovered from HRTV-DL1 infected ACAM34_UNSW cultures. Virus particles were incubated with host cells (or cell free media as control) for 5 min, 15min and 2 hours and the number of non-adsorbed free virus particles in the supernatant was determined by plaque assay. Graphs represent one of two biological replicates for ACAM34_UNSW and one of three biological replicates for UNSW_2.14, UNSW_3.3 and the media control. Error bars represent standard deviation from three independent experiments for UNSW_2.14, UNSW_3.3 and the media control, and two independent experiments for ACAM34_UNSW.

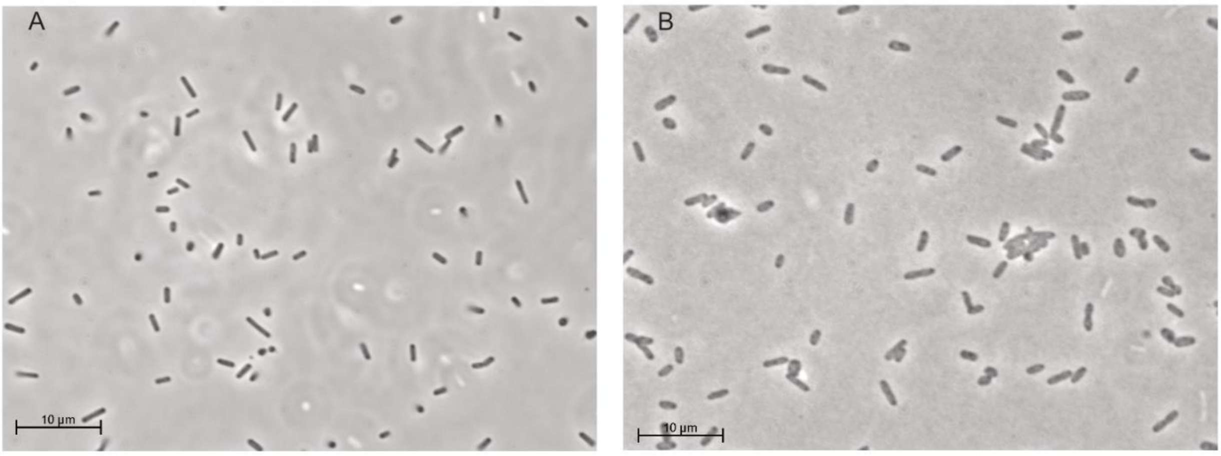

Figure S12. Cell morphology of *Hrr. lacusprofundi* UNSW_2.14. Light microscopic images of (A) ACAM34_UNSW parental strain in comparison with (B) UNSW_2.14. Pictures represent one of three biological replicates.

Supplementary Tables

Table S1. Annotation of HRTV-DL1 genome and identification of proteins in the virus particle. (Excel file)

Table S2. Proteins identified in purified virus particles. (Excel file)

Table S3. CRISPR spacer hits of Deep Lake haloarchaea against HRTV-DL1. (Excel file)

Table S4. Putative virus defense mechanisms in ACAM34_DSMZ. Output from PADLOC webserver [2] run on ACAM34_DSMZ. (Excel file)

Table S5. Differential expression of ACAM34_DSMZ genes under infection with HRTV-DL1. Differential expression at time point 1 (56 hours post infection), calculated with Geneious Prime® 2022.2.1, from 2 replicates uninfected and 3 replicates HRTV-DL1 infected. (Excel file)

Table S6. Differential expression of ACAM34_UNSW genes under infection with HRTV-DL1. Differential expression at time point 1 (56 hours post infection), calculated with Geneious Prime® 2022.2.1, from 2 replicates uninfected and 3 replicates HRTV-DL1 infected. (Excel file)

Table S7. Comparison of Expression levels of the S-layer gene (ACAM34_UNSW_01982) with Hlac_3088 (ACAM34_UNSW_02085) in ACAM34_UNSW. Expression values (FPKM) have been calculated with Geneious Prime® 2022.2.1, and averages from 2 replicates uninfected and 3 replicates HRTV-DL1 infected are presented.

Sample	ACAM34_UNSW_01982	ACAM34_UNSW_02085
**32 hours p.i. control**	496	401
**56 hours p.i. control**	544	267.5
**32 hours p.i. infected**	1876	294
**56 hours p.i. infected**	677.33	849

Table S8. Expression levels of HRTV-DL1 ORFs 56 hours p.i. FPKM values for HRTV-DL1 ORFs in three different replicates, and average of those, 56.1 hours post infection. Expression values (FPKM) have been calculated with Geneious Prime® 2022.2.1. (Excel file)

Table S9. Variant analysis on escape mutants. Variant analysis was performed with Geneious Prime® 2022.2.1. (Excel file)

Table S10: Primer Sequences (Excel file)

Table S11. Free virus particles in HRTV-DL1 infected escape mutants UNSW_2.14 and UNSW_3.3. Free virus particles calculated for 0.0001ul culture supernatant of HRTV-DL1 infected ACAM34_UNSW and escape mutants UNSW_2.14 and UNSW_3.3, as determined by plaque assay. Numbers are one representative from three biological replicates with standard deviation from the three independent experiments.

hours p. i.	ACAM34_UNSW	UNSW_2.14	UNSW_3.3
**41.8**	2700 ± 445.16	0.105 ± 0.035	0.095 ± 0.019
**89.7**	21345 ± 3552.08	0.099 ± 0.021	0.052 ± 0.008

Supplementary References

1.Tschitschko B, Erdmann S, DeMaere MZ, Roux S, Panwar P, Allen MA, et al. Genomic variation and biogeography of Antarctic haloarchaea. Microbiome. 2018;6(1):113. doi: 10.1186/s40168-018-0495-3.

2. Payne LJ, Meaden S, Mestre MR, Palmer C, Toro N, Fineran Peter C, et al. PADLOC: a web server for the identification of antiviral defence systems in microbial genomes. Nucleic acids research. 2022. doi: 10.1093/nar/gkac400.
